# Borates *vs*. aluminates: comparing the anion for lithium-ion batteries†

**DOI:** 10.1039/d4cc04812a

**Published:** 2024-11-22

**Authors:** Darren M. C. Ould, Megan E. Penrod, Jessica B. McConnell, Mohammed A. Zabara, Astrid H. Berge, Christopher A. O’Keefe, Andrew D. Bond, Svetlana Menkin, Clare P. Grey, Dominic S. Wright

**Affiliations:** a Yusuf Hamied Department of Chemistry, University of Cambridge Lensfield Road Cambridge CB2 1EW UK; b The Faraday Institution, Quad One, Harwell Science and Innovation Campus, Didcot OX11 ORA UK cpg27@cam.ac.uk dsw1000@cam.ac.uk

## Abstract

Lithium borate and aluminate salts bearing a hexafluoroisopropoxy ligand have been prepared and investigated for use in lithium-ion batteries and Cu‖Li cells. Lithium aluminate salts have poorer air tolerance but Li[Al(hfip)_4_] resulted in superior battery cycling, with lower overpotentials for plating and stripping in Cu‖Li cells.

## Abstract 2

Of a series of related borate and aluminate salts investigated, the unsolvated aluminate Li[Al(hfip)_4_] (hfip = (CF_3_)_2_CHO) resulted in superior battery cycling and is a promising electrolyte for next-generation lithium batteries.

## Graphical abstract

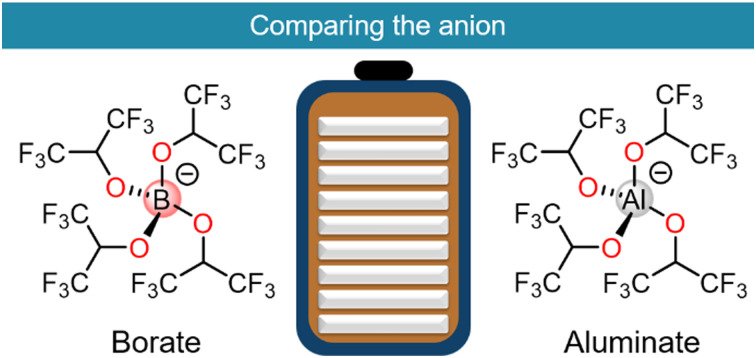

The electrolyte in a rechargeable battery is critical as it allows the movement of ions between the two electrodes during cycling and formation of interfaces. Electrolyte salts with a weakly-coordinating anion are known to improve the cyclability of lithium-ion batteries (LIBs) by offering high ionic conductivity,^1–3^ as a result of minimising ion pairing between the lithium cation and the anion. For LIBs, 1 M LiPF_6_ in carbonate solvents is widely used,^4,5^ since the use of this electrolyte at this salt concentration is a good compromise between ionic conductivity, electrochemical stability, safety and cost. However, LiPF_6_ is highly hygroscopic, having a low thermal decomposition temperature and reacting with electrolyte components to form highly toxic breakdown products.^6,7^

The use of weakly-coordinating quaternary borate and aluminate anions containing E(OR)_4_^−^ (E = B or Al, R = fluorinated ligand) as electrolyte salts has received significant attention. This can be attributed to the ability to tune the steric and electronic properties of the anion by ligand design, thus modifying the degree of cation–anion association. Of the different ligands trialled, hexafluoroisopropoxy [(hfip = OCH(CF_3_)_2_ (O^i^Pr^F^))] has proven effective, especially in the multivalent battery fields.^8,9^

In the magnesium-ion battery field Mg[B(hfip)_4_]_2_ is now established as a leading electrolyte salt.^10,11^ However, later the analogous aluminate salt Mg[Al(hfip)_4_]_2_ was shown to give better capacity utilisation and lower overpotentials in glyme-based solvents.^12^ A similar story has been observed when comparing the performance of [B(hfip)_4_]^−^ and [Al(hfip)_4_]^−^ anions in calcium-ion batteries, with recently reported Ca[Al(hfip)_4_]_2_ offering gains in ionic conductivity, plating and stripping efficiency and oxidative stability over its borate analogue.^13,14^

Our group has previously reported Na[B(hfip)_4_]·DME (DME = 1,2-dimethoxyethane) for use in sodium-ion batteries (NIBs), where greater capacity retention than NaPF_6_ was observed.^15^ Na[B(hfip)_4_]·3DME has also been studied in sodium-sulfur batteries.^16^ Interestingly, Na[Al(hfip)_4_]·DME was found to be a room-temperature solvated-ionic liquid, but gave poor capacity retention in NIBs.^17^

Li[B(hfip)_4_]·3DME has been studied as the salt for LIBs. It exhibits high oxidative stability and stable cycling in cells using a lithium metal anode and LiNi_0.8_Mn_0.1_Co_0.1_O_2_ (NMC811) cathode.^18^ However, Li[Al(hfip)_4_] salts have not been extensively studied in LIBs, although Li[Al(hfip)_4_] has been used in lithium-sulfur batteries,^19^ polymer electrolytes,^20^ and the fundamental electrochemical and transport properties have previously been reported.^21,22^

Herein, we report the synthesis of the lithium borate and lithium aluminate salts Li[B(hfip)_4_]·2DME (1a·2DME), Li[Al(hfip)_4_]·DME (1b·DME) and Li[Al(hfip)_4_] (1b). Fundamental properties of these salts were investigated, allowing comparisons between the anions to be made, and their suitability as electrolyte salts for battery use was explored.

The syntheses of the lithium borate and lithium aluminate salts were adapted from previous reports,^15,23^ involving the addition of pure lithium borohydride or lithium aluminium hydride to hexafluoroisopropanol (^i^Pr^F^OH) in 1,2-dimethoxyethane (DME) solvent (Scheme 1, top). After solvent removal and drying the solid lithium tetrakis(hexafluoroisopropoxy)borate salt at 85 °C under vacuum (1 × 10^−2^ mbar), the solution-state ^1^H NMR spectrum in CD_3_CN solvent revealed two solvating DME molecules per formula unit, Li[B(hfip)_4_]·2DME (1a·2DME, 52%) (Fig. S8.1.1–S8.1.5, ESI†). In contrast, for lithium tetrakis(hexafluoroisopropoxy)aluminate, ^1^H NMR spectroscopy revealed the presence of only one solvating DME molecule, Li[Al(hfip)_4_]·DME (1b·DME, 68%) (Fig. S8.1.6–S8.1.10, ESI†).

**Scheme 1:**
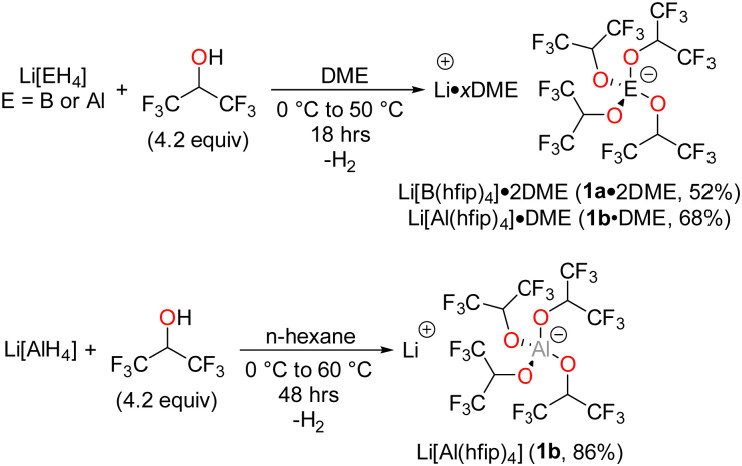
Top: synthetic procedure to produce Li[B(hfip)_4_]·2DME (1a·2DME) and Li[Al(hfip)_4_]·DME (1b·DME) using DME solvent. Bottom: synthetic procedure to produce Li[Al(hfip)_4_] (1b) using *n*-hexane solvent.

To produce salts without solvent coordination, the reactions were performed using *n*-hexane solvent (Scheme 1, bottom). The reaction with lithium borohydride and hexafluoroisopropanol gave an oil containing a mixture of species, as seen by ^11^B NMR spectroscopy. In contrast, the reaction with lithium aluminium hydride gave the desired complex Li[Al(hfip)_4_] (1b) as a white powder in good yield (86%) (Fig. S8.1.11–S8.1.15, ESI†). The lower basicity of BH_4_^−^ over AlH_4_^−^ may explain these differences in reactivity.^24^

Despite the observation of two DME molecules in Li[B(hfip)_4_]·2DME (1a·2DME) by ^1^H NMR spectroscopy in CD_3_CN solvent, single crystal X-ray diffraction showed that crystals grown by vacuum sublimation only have one DME donor, having an identical structure to that reported for Li[B(hfip)_4_]·DME (1a·DME).^9^ A very similar structure was obtained for the new complex Li[Al(hfip)_4_]·DME (1b·DME), where one DME molecule is coordinated to the lithium cation in a bidentate fashion (Fig. 1 and Fig. S2.2.1, ESI†). There is a noticeable difference in the coordination geometry of the Li^+^ cation in 1a·DME and 1b·DME, with the smaller B atom in the [B(hfip)_4_]^−^ anion in 1a·DME pulling the hexafluoroisopropoxy groups away from Li^+^ (see S2 in ESI†). It is plausible that this distortion leaves Li^+^ in 1a·DME more exposed to additional coordination by DME, as seen in the solution ^1^H NMR of the bulk sample. In 1b·DME, the lithium cation is more uniformly surrounded by the F atoms of the CF_3_ groups, which may inhibit further DME coordination (but the possibility that the second DME observed by NMR in 1a·2DME is lattice bound cannot be excluded). The solid-state structure of Li[Al(hfip)_4_] (1b) shows a dimeric structure with bridging Li^+^ cations. This crystal structure has also been reported previously.^25^

**Fig. 1:**
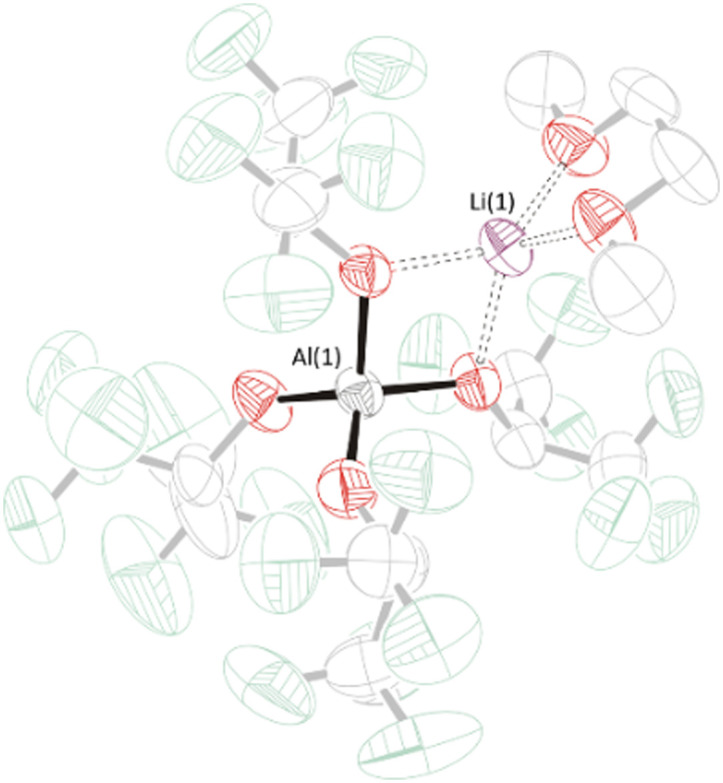
Solid-state structure of Li[Al(hfip)_4_]·DME (1b·DME). Displacement ellipsoids drawn at 50% probability, H-atoms removed, hfip ligand and DME molecule faded for clarity. Li: purple, Al: grey, O: red, F: green.

The air stabilities of Li[B(hfip)_4_]·2DME (1a·2DME) (2 DME as determined by ^1^H NMR spectroscopy on the bulk sample), Li[Al(hfip)_4_]·DME (1b·DME) and Li[Al(hfip)_4_] (1b) were assessed by leaving 0.1 mmol of each salt exposed to ambient air for 24 hours. An electrolyte salt with a high tolerance to air is advantageous as it facilitates convenient handling, transport and storage. Solution-state NMR spectroscopy in DMSO-d_6_ showed that no degradation of 1a·2DME had occurred. This was evident from retention of the signals at 1.5 ppm and −74.3 ppm in the ^11^B and ^19^F solution-state NMR spectra, respectively, which correspond to the intact [B(hfip)_4_]^−^ anion (no other signals being observed, Fig. S8.2.1–S8.2.8, ESI†). Conversely, for the air exposed lithium aluminate salts 1b·DME and 1b, there was obvious decomposition. This was seen with the formation of insoluble products in DMSO-d_6_, unlike the pristine salts which have high solubility in this solvent. Solid-state NMR (ssNMR) spectroscopy confirmed the almost complete degradation of the [Al(hfip)_4_]^−^ anion (Fig. S8.2.9–S8.2.11, ESI†). This was most evident from the ^27^Al ssNMR spectrum, which showed the formation of a new resonance at 6.9 ppm (Fig. 8.2.11, ESI†), indicative of a six-coordinate aluminium (likely a water complex, *cf*. 60.0 ppm in CD_3_CN for pristine 1b·DME and 1b). The differences in the air tolerance between the borate and aluminate salts is mainly due to the greater polarity of the Al–O bonds and the coordinatively saturated boron atom of the [B(hfip)_4_]^−^ anion.

Electrolyte salts with high thermal stability are beneficial as they potentially enable high-temperature battery cycling. The thermogravimetric analysis (TGA) profiles for 1a·2DME, 1b·DME and 1b are similar in appearance and show a one-step thermal decomposition process (Fig. S3.2.1–S3.2.4, ESI†). The lithium aluminate salt 1b·DME has the highest thermal decomposition temperature, with an onset temperature of 182 °C. 1a·2DME and 1b have slightly lower onset temperatures for decomposition of 164 and 166 °C, respectively.

The electrochemical properties of these salts were investigated by firstly measuring their bulk conductivities in 1 M solutions in ethylene carbonate: ethyl methyl carbonate (EC : EMC 3 : 7 v/v) (see S4.5, ESI†). The three electrolytes gave similar conductivities at 20 °C, with 1 M Li[Al(hfip)_4_]·DME (1b·DME) in EC : EMC giving the highest value of 7.2 ± 0.2 mS cm^−1^ (Fig. 2). The values for Li[Al(hfip)_4_] (1b) and Li[B(hfip)_4_]·2DME (1a·2DME) were the same, at 6.9 ± 0.2 mS cm^−1^. These conductivity values are similar to 1 M LiPF_6_ in EC : EMC (3 : 7 v/v) (LP57) (8.0 ± 0.3 mS cm^−1^). The slightly higher conductivity of the aluminate salt 1b·DME is consistent with findings on analogous calcium salts, which was suggested to result from the lower tendency of the [Al(hfip)_4_]^−^ anion to form contact ion-pairs and aggregates in solution.^14^

**Fig. 2:**
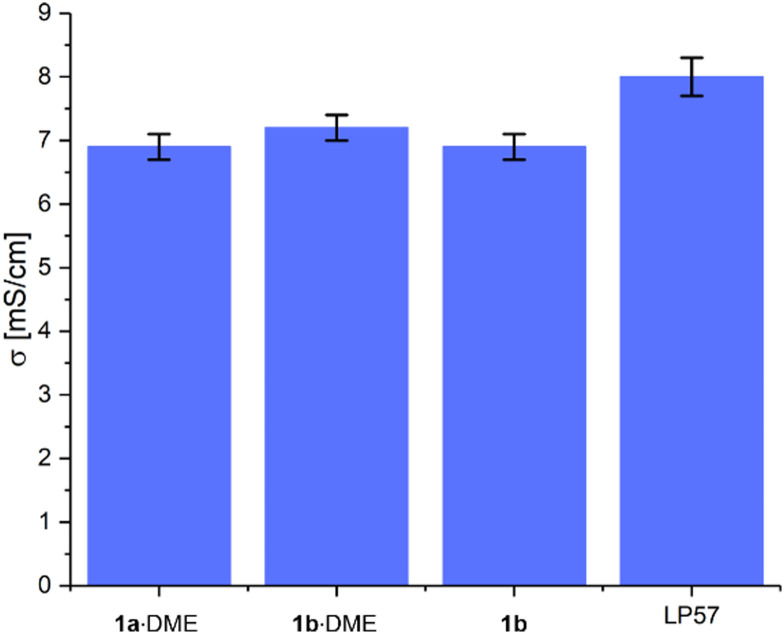
Bulk conductivities of 1 M Li[B(hfip)_4_]·DME (1a·2DME), 1 M Li[Al(hfip)_4_]·DME (1b·DME), 1 M Li[Al(hfip)_4_] (1b) and 1 M LiPF_6_ (LP57) in EC : EMC (3 : 7 v/v) solvent, measured at 20 °C. Error bars correspond to error in the EIS fitting.

Dynamic viscosity measurements were recorded to help rationalise transport. In 1 M solutions in EC : EMC 3 : 7 v/v at 28 °C, Li[B(hfip)_4_]·2DME (1a·2DME) and Li[Al(hfip)_4_]·DME (1b·DME) were less viscous than unsolvated Li[Al(hfip)_4_] (1b), with 1a·2DME having the lowest viscosity [2.7 cP (1a·2DME), 2.8 (1b·DME) and 3.2 cP (1b)] (Fig. S5.2, ESI†). All three electrolytes have lower viscosities than LP57 (3.7 cP). The diffusion coefficients of these electrolyte solutions were determined by ^1^H NMR diffusion-ordered spectroscopy (DOSY) and support the viscosity measurements, with the diffusion coefficients of EC solvent molecules being larger for the electrolytes containing DME, 1a·2DME and 1b·DME, compared to 1b (Table S6.2, ESI†). In addition, using ^19^F DOSY the diffusion coefficient of the [B(hfip)_4_]^−^ anion in 1a·2DME was marginally higher than for the [Al(hfip)_4_]^−^ anion in either 1b·DME or 1b, as expected on account of the smaller size of the borate anion (Table S6.2, ESI†).

The electrochemical stability windows (ESWs) of 1 M solutions of Li[B(hfip)_4_]·2DME (1a·2DME), Li[Al(hfip)_4_]·DME (1b·DME) and Li[Al(hfip)_4_] (1b) in EC : EMC (3 : 7 v/v) were determined by cyclic voltammetry (CV). This was performed using a two-electrode cell with either a copper or aluminium working electrode (WE) and lithium metal counter electrode; this experiment allowed for a direct comparison of the ESW of the different electrolytes to be made. Copper and aluminium were chosen as they are commonly used as the current collector for the anode and cathode, respectively, in LIBs. The reductive stabilities of the electrolytes were determined from the CVs using copper as the WE, which showed irreversible peaks for the solid–electrolyte interphase (SEI) formation below 1.5 V *vs*. Li/Li^+^ for all electrolytes, with similar current magnitudes (Fig. S4.2.1, ESI†).^26^ Oxidative stability was evaluated using aluminium as the WE, which revealed higher oxidative stabilities for both 1a·2DME and 1b than LiPF_6_, as can be seen in Fig. 3 (top). Passivation, in the form of AlF_3_-containing protective films, are known to form when using LiPF_6_ on aluminium foils.^27^ The passivation capability of 1a·2DME and 1b electrolytes was not measured in this work. However, previous work by MacFarlane, Kar *et al*. has revealed the formation of AlF_3_ when using 0.5 M 1a· 3DME in ethylene carbonate: dimethyl carbonate at 5 V on aluminium.^18^ Understanding the passivation mechanism of these electrolytes is the focus of our future work.

**Fig. 3:**
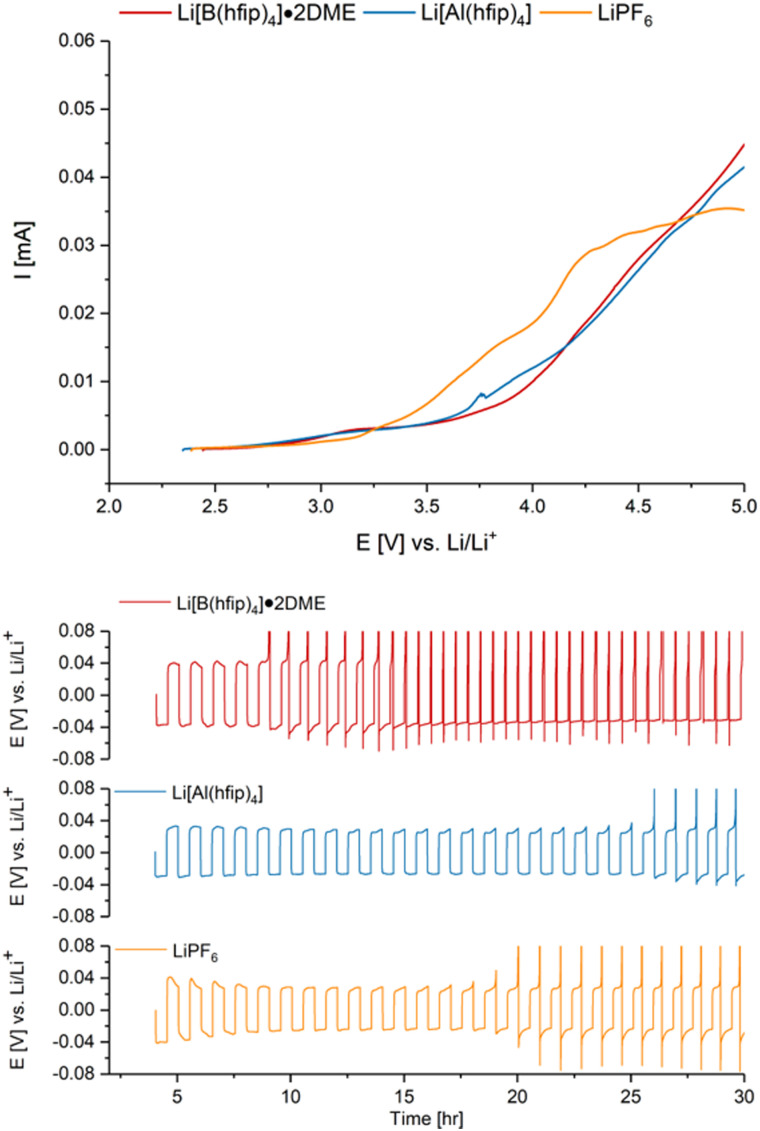
Top: Linear voltammetry results using a two-electrode cell, aluminium working electrode and lithium metal counter electrode. First cycle measured at a scan rate of 1 mV s^−1^ from open circuit voltage to 5 V. Bottom: Lithium *vs*. copper cell cycling using 1 M Li[B(hfip)_4_]·DME (1a·2DME) (red), 1 M Li[Al(hfip)_4_] (1b) (blue) and 1 M LiPF_6_ (orange) in EC : EMC (3 : 7 v/v) solvent as electrolytes. Current density 0.4 mA cm^−2^ and aerial capacity of 0.2 mA h cm^−2^.

Galvanostatic cycling on Cu‖Li cells was performed to evaluate the overpotentials associated with lithium plating and stripping and to assess the reversibility of these processes for each electrolyte (Fig. 3 bottom and Fig. S4.4.1–S4.4.5, ESI†). The 1 M solution of Li[Al(hfip)_4_] (1b) in EC : EMC exhibited the lowest overpotential, along with improved cycling stability. In contrast, Li[B(hfip)_4_]·2DME (1a·2DME) demonstrated the highest overpotential and poorest cycling stability, with 1 M LiPF_6_ displaying intermediate behaviour between the two. These findings highlight the superiority of 1b in lithium plating/stripping reactions, which can be attributed to enhanced reaction kinetics and likely improved SEI formation. This is further supported by electrochemical impedance spectroscopy (EIS) measurements (Fig. S4.3.1, ESI†) which showed two semicircles related to SEI and plating/stripping charge-transfer. The charge-transfer resistance was the lowest for 1b (≈16 Ω), which increases for 1b·DME (≈22 Ω), and is the highest for 1a·2DME (≈35 Ω). Equivalently, the SEI-related region showed higher SEI resistance for 1a·2DME, suggesting more hindrance of Li-ion movement through the formed SEI. This resistance is lower for the aluminate electrolytes 1b·DME and 1b, regardless of the presence of DME.

Lastly, the 1 M solutions of Li[B(hfip)_4_]·2DME (1a·2DME), Li[Al(hfip)_4_]·DME (1b·DME) and Li[Al(hfip)_4_] (1b) in EC : EMC were used as electrolytes in LIBs. For this, coin cells were constructed using NMC811 *vs*. graphite as the active cathode and anode materials, respectively. The cells were cycled at a 1C rate in the voltage range of 2.5–4.2 V. All three electrolytes were cycled for the 40-cycle duration, where differences were observed. The most stable cycling occurred for 1b, as when using 1a·2DME and 1b·DME, lower initial capacities and poorer capacity retentions were observed. After 40 cycles, the capacity retention using 1b was 80%, compared to 52% and 51% for 1a·2DME and 1b·DME, respectively (Fig. 4). Moreover, the Coulombic efficiencies using 1a·2DME and 1b·DME started low and only reached 92.9% and 85.2%, respectively, after the 40 cycles (Fig. S4.5.1, ESI†). Thus, showing the detrimental role of DME in the electrolyte salt. Compared to using LP57, 1b in EC : EMC cycled with slightly lower capacity.

**Fig. 4:**
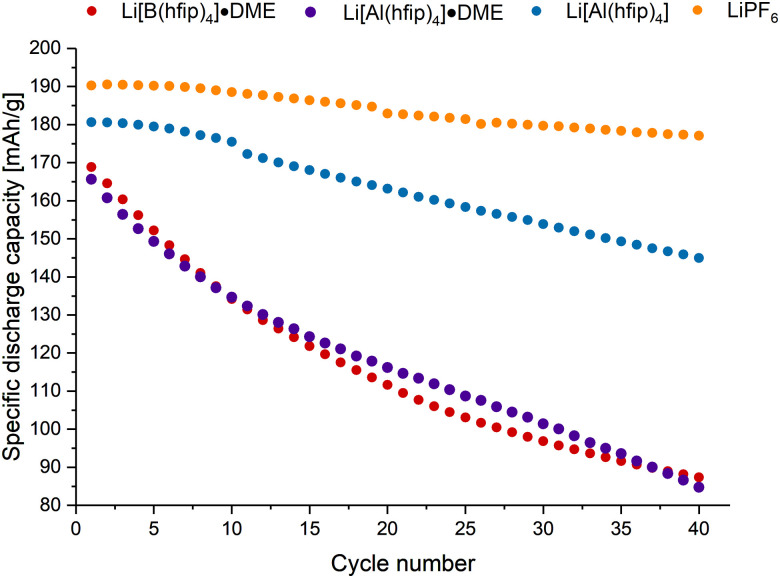
Discharge gravimetric capacity *vs*. cycle number for 1 M Li[B(hfip)_4_]·2DME (1a·2DME) (red), 1 M Li[Al(hfip)_4_]·DME (1b·DME) (purple), 1 M Li[Al(hfip)_4_] (1b) (blue) and 1 M LiPF_6_ (LP57) (orange) in EC : EMC electrolytes. Approximate constant current rate of 1C for charge and discharge using cell voltage limits of 4.2 and 2.5 V.

In conclusion, while Li[B(hfip)_4_]·2DME (1a·2DME) was found to have the greatest air tolerance, use of Li[Al(hfip)_4_] (1b) as a battery electrolyte in both lithium-ion and Cu‖Li cells was more stable than 1a·2DME. Investigations in Cu‖Li cells revealed that 1 M 1b has lower overpotentials for plating and stripping and improved cycling stability compared to 1 M 1a·2DME and 1 M LiPF_6_. Thus, this work encourages the use of lithium aluminate salts over their more commonly studied borate counterparts.

DMCO, MEP, JBM, MAZ, CPG and DSW would like to thank The Faraday Institution for funding this work; FIRG064 and FIRG060. A. H. B. thanks the EU for funding Advanced EU ERC grant (EC H2020 835073). S. M. gratefully acknowledges funding by the Royal Society University Research Fellowship (URF\R1\231513).

## Conflicts of interest

There are no conflicts to declare.

## Supplementary Material

CC-061-D4CC04812A-s001

CC-061-D4CC04812A-s002

## Data Availability

Data for this paper, including synthetic procedures, NMR spectra, summary of X-ray crystallographic data collections and refinements, thermogravimetric analysis, and electrochemical analysis are available in the supporting information [https://doi.org/10.1039/D4CC04812A]. The X-ray crystallographic data has been deposited with the Cambridge Crystallographic Data Base (https://www.ccdc.cam.ac.uk/structures/).
