# Supplementary material for: Borates vs. aluminates: comparing the anion for lithium-ion batteries
Source: Chem Commun (Camb). 2024 Nov 22;61(1):129–32. doi: 10.1039/d4cc04812a (PMC11609998; doi:10.1039/d4cc04812a)

Supplementary Information (SI) for ChemComm.
This journal is © The Royal Society of Chemistry 2024

Supplementary Information for:

Borates vs. Aluminates: Comparing the Anion for Lithium-Ion Batteries

Darren M. C. Ould,[a,b] Megan E. Penrod,[a,b] Jessica McConnell,[a,b] Mohammed A. Zabara,[a,b] Astrid H. Berge,[a] Christopher A. O'Keefe,[a] Andrew D. Bond,[a] Svetlana Menkin,[a,b] Clare P. Grey[a,b]* and Dominic S. Wright[a,b]*

[a] Yusuf Hamied Department of Chemistry, University of Cambridge, Lensfield Road, Cambridge, CB2 1EW, U.K.

[b] The Faraday Institution, Quad One, Harwell Science and Innovation Campus, Didcot, OX11 ORA, U.K.

Contents

S1 Synthesis of lithium borate and lithium aluminate salts. 2
S2 Single crystal X-ray diffraction. 5
S3 Thermal measurements. 10
S4 Electrochemistry measurements. 12
S5 Viscosity measurements. 22
S6 NMR measurements. 23
S7 References. 24
S8 NMR spectra. 25

S1 Synthesis of lithium borate and lithium aluminate salts.

S1.1 General Experimental

Unless stated otherwise, all reactions were carried out under an atmosphere of dinitrogen using standard Schlenk and glove box (under argon; Saffron, Alpha model) techniques. 1,2-dimethoxyethane (DME) solvent was dried over 4 Å activated molecular sieves for 24 hours and stored in an ampoule fitted with a Teflon valve under a dinitrogen atmosphere. Hexane and diethyl ether were collected freshly distilled over sodium-potassium amalgam before use. Deuterated solvents were dried over 4 Å activated molecular sieves and stored in an argon filled glovebox. 1,1,1,3,3,3-hexafluoro-2-propanol was purchased from Fluorochem, dried over 4 Å activated molecular sieves for 24 hours and stored over these molecular sieves in an ampoule fitted with a Teflon valve under a dinitrogen atmosphere. Lithium borohydride and lithium aluminium hydride were purchased from Sigma-Aldrich, crystallised from diethyl ether and dried at 90 °C *in vacuo* (1×10^{-2} mbar) for two hours. Ethylene carbonate: ethyl methyl carbonate (EC:EMC 3:7 v/v) was purchased from Solvionic and dried over 4 Å activated molecular sieves for 24 hours before use.

^{1}H, $^{13}C\{^{1}H\}$, ^{19}F, ^{11}B and ^{27}Al solution-state NMR spectra were recorded at 298.0 K on a Bruker 400 MHz AVIII HD Smart Probe spectrometer. Chemical shifts are expressed as parts per million (ppm, δ) and are referenced to CD_3CN (1.95/118.26 ppm) and $(CD_3)_2SO$ (2.50/39.52 ppm) as internal standards. Multinuclear NMR spectra were referenced to $BF_3{\cdot}Et_2O/CDCl_3$ (^{11}B), $CFCl_3$ (^{19}F) and $AlCl_3{\cdot}6H_2O/D_2O$ (^{27}Al). The description of signals includes s = singlet, d = doublet, t = triplet, q = quartet, q = quintet and m = multiplet. All coupling constants are absolute values and are expressed in Hertz (Hz). High-resolution mass spectra (HRMS) were collected by the School of Chemistry in University of Cambridge using a Waters Xevo G2-S QTOF mass spectrometer in negative mode.

S1.2 Synthesis of lithium borate salts.

Synthesis of lithium tetrakis(hexafluoroisopropoxy)borate, *Li[B(hfip)$_4$]·2DME* (**1a**·2DME)

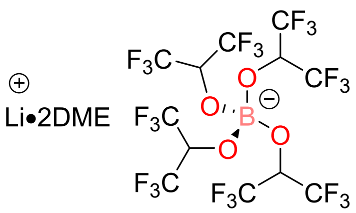

A Schlenk tube was charged with purified lithium borohydride (405 mg, 18.6 mmol, 1.0 equiv) and 1,2-dimethoxyethane (DME, 30 ml). The solution was cooled to 0 °C and 1,1,1,3,3,3-hexafluoro-2-propanol (8.2 ml, 78.1 mmol, 4.2 equiv) was added dropwise. The evolution of dihydrogen was immediately observed with effervescence. The reaction was left to stir at 0 °C for 30 minutes, before slowly warming to room temperature. The solution was then heated to 50 °C and left to stir for 18 hours. After heating, the reaction was left to cool to ambient temperature and the solvent was removed *in vacuo* (1×10^{-2} mbar), giving a white powder. This was then heated *in vacuo* at 85 °C for three hours, where partial sublimation of the product on the side of the Schlenk

tube was observed. ^{1}H NMR spectroscopy revealed the product exists as a DME solvated adduct, with 2 DME molecules, Li[B(hfip)$_4$]·2DME. Yield: 8.38 g, 9.68 mmol, 52%. Note the yield was calculated from the molecular weight of Li[B(hfip)$_4$]·2DME, as found by ^{1}H NMR spectroscopy, and not Li[B(hfip)$_4$]·DME as determined by single-crystal X-ray diffraction.

^{1}H NMR (400 MHz, CD_3CN, 295 K) δ/ppm: 4.72 (br s, 4 H, CH), 3.48 (s, 8H, DME–CH_2), 3.31 (s, 12H, DME–CH_3). **^{13}C{^{1}H} NMR** (101 MHz, $(CD_3)_2SO$, 295 K) δ/ppm: 126.7–118.2 (m, CF_3), 71.1 (s, DME–CH_2) 69.3–68.3 (m, CH), 58.0 (s, DME–CH_3). **^{11}B NMR** (128 MHz, CD_3CN, 295 K) δ/ppm: 1.2 (quin, $^{3}J_{BH}$ = 3.1 Hz). **^{19}F NMR** (376 MHz, CD_3CN, 295 K) δ/ppm: −75.4 (s). **^{7}Li NMR** (155 MHz, $(CD_3)_2SO$, 295 K) δ/ppm: −1.79 (s). **HRMS** (ASAP$^-$) *m/z* calculated for $[M]^-$ $[C_{12}H_4BO_4F_{24}]^-$: 678.9819 found: 678.9939. Spectroscopic analyses of the anion in agreement with literature values.[1]

*Synthesis of lithium tetrakis(*hexafluoroisopropoxy*)aluminate, Li[Al(hfip)$_4$]·DME* (**1b**·DME)

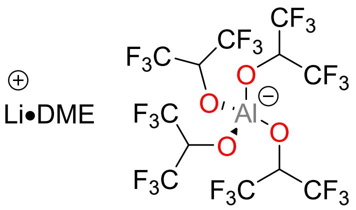

A Schlenk tube was charged with purified lithium aluminium hydride (345 mg, 9.09 mmol, 1.0 equiv) and 1,2-dimethoxyethane (DME, 30 ml). The solution was cooled to 0 °C and 1,1,1,3,3,3-hexafluoro-2-propanol (4.0 ml, 38.2 mmol, 4.2 equiv) was added dropwise. The evolution of dihydrogen was immediately observed with effervescence. The reaction was left to stir at 0 °C for 30 minutes, before slowly warming to room temperature. The solution was then heated to 50 °C and left to stir for 18 hours. After heating, the reaction was left to cool to ambient temperature and the solvent was removed *in vacuo* (1×10^{-2} mbar), giving a white powder. This was then heated *in vacuo* at 85 °C for three hours, where partial sublimation of the product on the side of the Schlenk tube was observed. ^{1}H NMR spectroscopy revealed the product exists as a DME solvated adduct with 1 DME molecule, Li[Al(hfip)$_4$]·DME. Yield: 4.92 g, 6.21 mmol, 68%.

^{1}H NMR (400 MHz, CD_3CN, 295 K) δ/ppm: 4.58–4.55 (m, 4 H, CH), 3.49 (s, 4H, DME–CH_2), 3.32 (s, 6H, DME–CH_3). **^{13}C{^{1}H} NMR** (101 MHz, CD_3CN, 295 K) δ/ppm: 129.1–119.2 (m, CF_3), 72.5–71.2 (m, CH), 72.1 (s, DME–CH_2), 59.1 (s, DME–CH_3). **^{27}Al NMR** (104 MHz, CD_3CN, 295 K) δ/ppm: 60.0 (s). **^{19}F NMR** (376 MHz, CD_3CN, 295 K) δ/ppm: −77.7 (s). **^{7}Li NMR** (155 MHz, CD_3CN, 295 K) δ/ppm: −2.49 (s). **HRMS** (ASAP$^-$) *m/z* calculated for $[M]^-$ $[C_{12}H_4AlO_4F_{24}]^-$: 694.9542 found: 694.9531. Spectroscopic analyses of the anion in agreement with literature values.[2]

*Synthesis of lithium tetrakis(*hexafluoroisopropoxy*)aluminate, Li[Al(hfip)$_4$]* (**1b**)

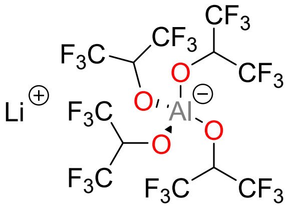

A Schlenk tube was charged with purified lithium aluminium hydride (616 mg, 16.2 mmol, 1.0 equiv) and hexane (40 ml). The solution was cooled to 0 °C and 1,1,1,3,3,3-hexafluoro-2-propanol (7.2 ml, 68.2 mmol, 4.2 equiv) was added dropwise. The evolution of dihydrogen was immediately observed with effervescence. The reaction was left to stir at 0 °C for one hour, before slowly warming to room temperature. The solution was then heated to 50 °C and left to stir for 18 hours. A white precipitate was observed at this point. After heating, the reaction was left to cool to ambient temperature and the solvent was removed by filtration using a filter cannula. This resulting white powder was then heated *in vacuo* (1 × 10^{-2} mbar) at 90 °C for three hours, giving the product Li[Al(hfip)$_4$]. Yield: 9.807 g, 14.0 mmol, 86%.

^{1}H NMR (400 MHz, CD_3CN, 295 K) δ/ppm: 4.58–4.55 (m, 4 H, CH). **^{13}C{^{1}H} NMR** (101 MHz, CD_3CN, 295 K) δ/ppm: 124.0 (quart, $\underline{C}F_3$, $^1J_{FC}$ = 284 Hz), 72.4–71.1 (m, CH). **^{27}Al NMR** (104 MHz, CD_3CN, 295 K) δ/ppm: 60.0 (s). **^{19}F NMR** (376 MHz, CD_3CN, 295 K) δ/ppm: −77.7 (s). **^{7}Li NMR** (155 MHz, CD_3CN, 295 K) δ/ppm: −2.63 (s). **HRMS** (ASAP$^-$) *m/z* calculated for [M]$^-$ [$C_{12}H_4AlO_4F_{24}$]$^-$: 694.9542 found: 694.9531. Spectroscopic analyses of the anion in agreement with literature values.[2]

S2 Single crystal X-ray diffraction.

S2.1 General X-ray diffraction experimental

Single-crystal X-ray data for Li[B(hfip)$_4$]·DME (**1a**·DME), Li[Al(hfip)$_4$]·DME (**1b**·DME) and Li[Al(hfip)$_4$] (**1b**) were collected on a Bruker D8-QUEST diffractometer, equipped with an Incoatec IμS Cu microsource (λ = 1.5418 Å) and a PHOTON-III detector operating in shutterless mode. The crystal was mounted on a MiTeGen crystal mount using inert polyfluoroether oil and the analysis was carried out under an Oxford Cryosystems open-flow N_2 Cryostream. The control and processing software was Bruker *APEX5*. Diffraction images were integrated using *SAINT* in *APEX5*, and a multi-scan correction was applied using *SADABS*. The final unit-cell parameters were refined against all reflections. Structures were solved using *SHELXT* and refined using *SHELXL*.

The structures of (**1a**·DME) and (**1b**) are identical to those published previously:

- (**1a**·DME) Li[B(hfip)$_4$]·DME: Mandai, Naya, Masu, *J. Phys. Chem. C* (2023), **127**, 7987–7997.
- (**1b**) Li[Al(hfip)$_4$]: Ivanova, Nolan, Kobayashi, Miller, Anderson, Strauss, *Chem.-Eur. J.* (2001), **7**, 503–510. CSD: MIDJAK.

Our redeterminations are summarised in Table S2.3. For both structures, there is no significant difference compared to the previously determined structure.

S2.2. Crystal structures

Compounds (**1a**·DME) and (**1b**·DME) crystallise with similar structures. The molecular units are illustrated below:

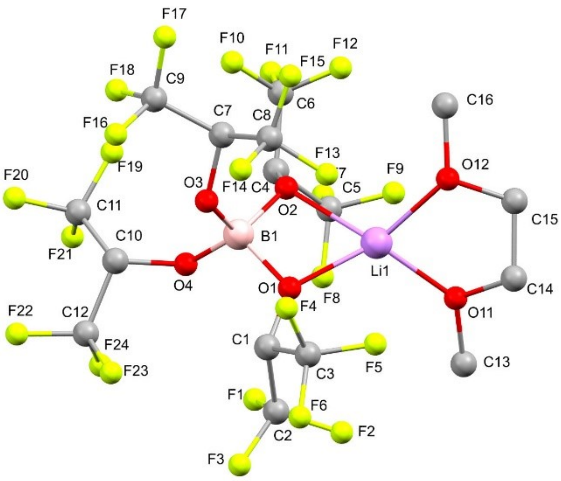

(**1a**) Li[B(hfip)$_4$]·DME (Li: τ_4' = 0.52)

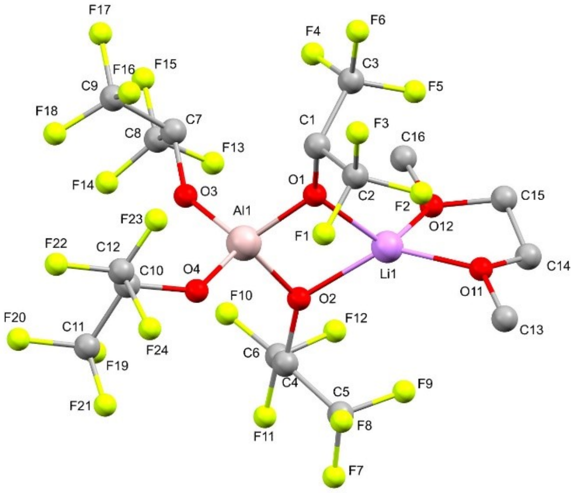

(**1b**) Li[Al(hfip)$_4$]·DME (Li: τ_4' = 0.64)

The coordination geometry of Li$^+$ is intermediate between square planar and tetrahedral, while it is closer to tetrahedral in **1b**·DME (quantified by the parameter τ_4'). Overlaying the two molecular structures so that the Al/B and Li atoms are roughly superimposed shows that the smaller B atom in **1a**·DME (red) pulls the CF_3 groups further from the Li$^+$ atom compared to **1b**·DME (blue). This distortion probably contributes to the different Li$^+$ coordination geometry in the two structures and is likely to impact the propensity for the complex to coordinate an additional DME ligand at Li$^+$, as seen in solution for **1a**·DME.

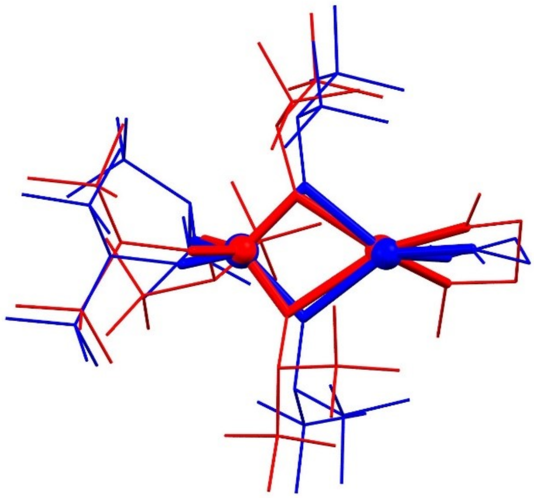

References for the τ_4' parameter:

- http://kchn.pg.gda.pl/geom/?p=home
- Okuniewski, D. Rosiak, J. Chojnacki, B. Becker (2015). *Polyhedron* **90,** 47–57.
- D. Rosiak, A. Okuniewski, J. Chojnacki (2018). *Polyhedron* **146**, 35–41.

Structure (**1b**) comprises two Li[Al(hfip)$_4$] units linked into a dimer. In the absence of the coordinating DME ligands, Li$^+$ expands its coordination sphere by associating with a neighbouring Li[Al(hfip)$_4$] unit. The coordination geometry around Li$^+$ comprises four O atoms from the two AlO_4 tetrahedra, plus four further clear Li···F contacts in the range 2.413(6)–3.025(7) Å.

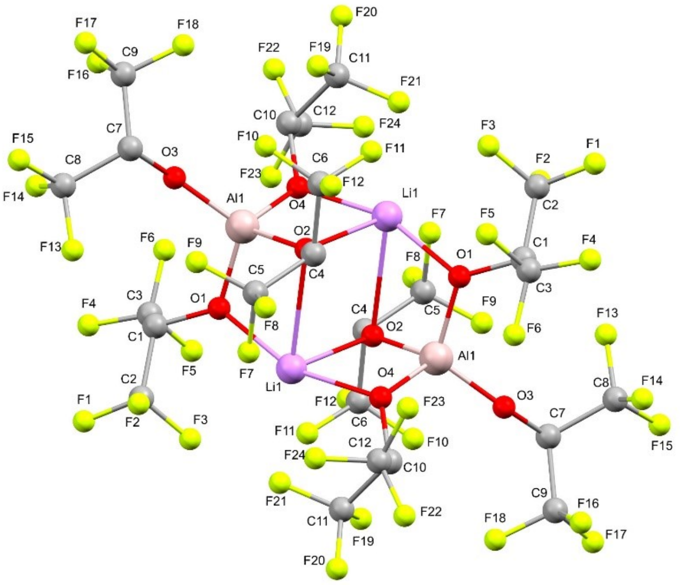

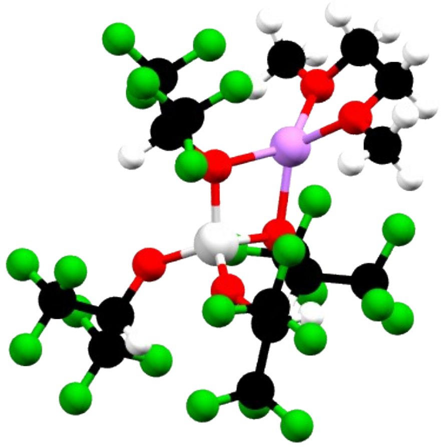

Figure S2.2.1 Solid-state structure of Li[Al(hfip)$_4$]·DME (1b•DME) as ball and stick model. Li: purple, Al: grey, O: red, F: green, C: black.

S2.3. Summary of crystal structure and refinement details

	(**1a**·DME)	(**1b**·DME)	(**1b**)
	Li[B(hfip)$_4$]·DME	Li[Al(hfip)$_4$]·DME	Li[Al(hfip)$_4$]
CCDC number	2382827	2360443	2382828
Cambridge data number	DW_B1_0551	DW_B1_0553	DW_B1_0552
Chemical formula	$C_{16}H_{14}BF_{24}LiO_6$	$C_{16}H_{14}AlF_{24}LiO_6$	$C_{24}H_8Al_2F_{48}Li_2O_8$
Formula weight	776.02	792.19	1404.14
Temperature / K	180(2)	200(2)	200(2)
Crystal system	triclinic	triclinic	triclinic
Space group	P –1	P –1	P –1
a / Å	10.3513(4)	10.1045(6)	10.1467(3)
b / Å	10.4771(4)	10.3479(6)	10.5435(3)
c / Å	13.3717(5)	15.5279(9)	11.6900(3)
α / °	79.860(2)	78.701(3)	76.185(2)
β / °	78.622(2)	73.361(3)	80.386(2)
γ / °	76.509(2)	79.766(3)	65.157(2)
Unit-cell volume / Å^3	1369.53(9)	1512.65(16)	1098.90(6)
Z	2	2	1
Calc. density / g cm^{-3}	1.882	1.739	2.122
F(000)	764	780	680
Radiation type	Cu Kα	Cu Kα	Cu Kα
Absorption coefficient / mm^{-1}	2.230	2.307	2.998
Crystal size / mm^3	0.12 x 0.10 x 0.04	0.16 x 0.16 x 0.16	0.10 x 0.08 x 0.06
2θ range / °	6.81–136.60	6.01–136.74	7.81–136.67
Completeness to max 2θ	0.991	0.987	0.994
No. of reflections measured	19588	22760	18345
No. of independent reflections	4993	5486	3994
R(int)	0.0430	0.0589	0.0548
No. parameters / restraints	435 / 0	435 / 144	379 / 0
Final R1 values (I > 2σ(I))	0.0385	0.0819	0.0486
Final wR(F^2) values (all data)	0.0951	0.2494	0.1224
Goodness-of-fit on F^2	1.025	1.079	1.043
Largest difference peak & hole / e Å^{-3}	0.262, –0.280	0.565, –0.437	0.428, –0.354

S3 Thermal measurements.

S3.1 Thermal stability general experimental.

Thermogravimetric analysis (TGA): TGA data was recorded with a Mettler Toledo TGA / DSC 2 Star^{ed} system equipped with a Huber minichiller. A few milligrams of sample were taken out of the argon-filled glovebox and immediately transferred to the TGA heating chamber to minimise air exposure. All the measurements were performed from 25 °C to 600 °C with a heating rate of 10 °C min^{-1} and under nitrogen flow.

S3.2 Thermal stability plots.

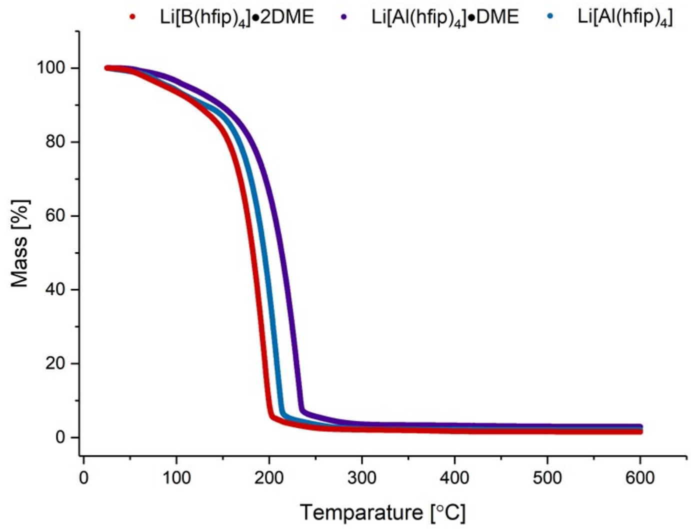

Figure S3.2.1 TGA curves for salts $Li[B(hfip)_4]\cdot 2DME$ (red), $Li[Al(hfip)_4]\cdot DME$ (purple) and $Li[Al(hfip)_4]$ (blue). Heating rate of 10 °C min^{-1} and under a nitrogen flow.

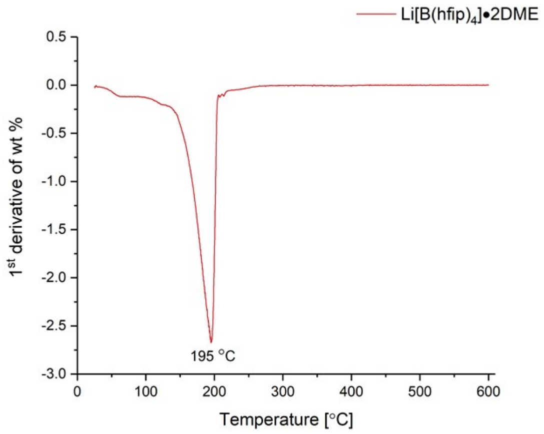

Figure S3.2.2 1st derivative of the Weight *vs*. Temperature plot for $Li[B(hfip)_4]\cdot 2DME$ electrolyte salt, showing the inflection point temperature.

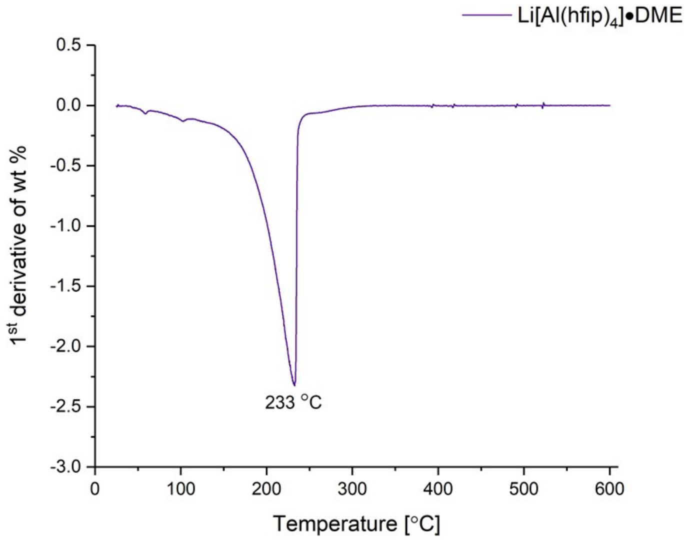

Figure S3.2.3 1st derivative of the Weight *vs*. Temperature plot for Li[Al(hfip)$_4$]·DME electrolyte salt, showing the inflection point temperature.

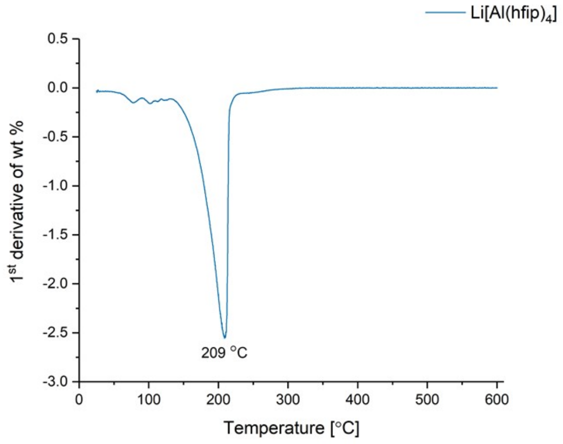

Figure S3.2.4 1st derivative of the Weight *vs*. Temperature plot for Li[Al(hfip)$_4$] electrolyte salt, showing the inflection point temperature.

S4 Electrochemistry measurements.

S4.1 Conductivity measurement experimental.

Solution conductivity measurements were made in a TSC 70 Closed cell from RHD instruments. 70 µl of each liquid was filled into the cell and sealed inside an argon-filled glovebox. Impedance spectra were measured using a PalmSens4 or Biologic potentiostat, with an applied voltage amplitude of 10 mV and frequencies between 1 MHz and 1 Hz. The impedance spectra were fitted using the equivalence circuit R+Q, and the solution conductivity was found by taking the reciprocal of the R component, multiplied by the cell constant.

The cell constant was determined using a 1413 µS cm^{-1} conductivity standard solution from Hanna Instruments. 70 µl of the solution was filled into the cell, which was sealed and placed in an incubator held at 25°C. An impedance spectrum was measured as above. The spectrum was fitted with a Q+R/Q circuit and the measured resistance was multiplied by 1413 µS cm^{-1} to determine the cell constant, which was found to be 6.1 ± 0.1cm^{-1}.

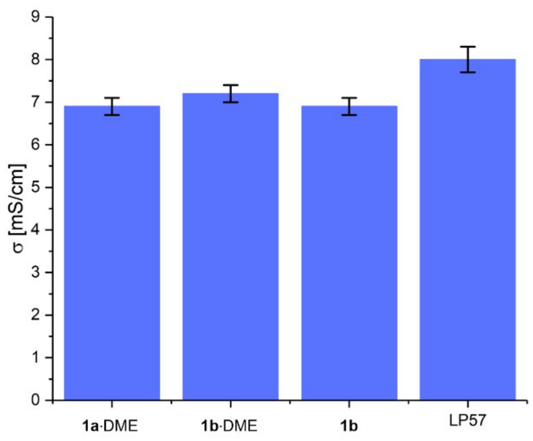

Figure 4.1.1 Bulk conductivities of 1 M Li[B(hfip)$_4$]·2DME (**1a**·2DME), 1 M Li[Al(hfip)$_4$]·DME (**1b**·DME), 1 M Li[Al(hfip)$_4$] (**1b**) and 1 M $LiPF_6$ (LP57) in EC:EMC (3:7 v/v) solvent, measured at 20 °C. Error bars correspond to error in the EIS fitting.

S4.2 Cyclic voltammetry

Cyclic Voltammetry (CV) measurements used a two-electrode cell assembly in coin cells (see lithium-ion cycling section) with a glass fibre separator and 100 µl of electrolyte. Either a copper or aluminium working electrode and lithium metal counter electrode were used. Measurements were recorded on a Biologic VSP potentiostat/galvanostat and started from the open circuit voltage (≈2.5 V *vs*. Li/Li^+) to 0.0 V *vs*. Li/Li^+ for copper and open circuit voltage to 5.0 V *vs*. Li/Li^+ for aluminium, with a sweep rate of 1 mV/s.

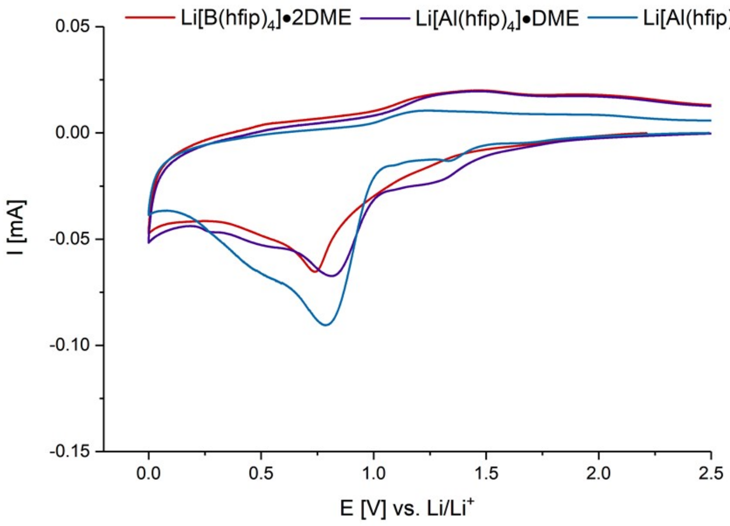

Figure S4.2.1 Cyclic voltammetry using a two-electrode cell, copper working electrode and lithium metal counter electrode. First cycle measured at a scan rate of 1 mV/s. Electrolytes are 1 M $Li[B(hfip)_4]\cdot 2DME$ (red) (**1a**·2DME), 1 M $Li[Al(hfip)_4]\cdot DME$ (purple) (**1b**·DME) and 1 M $Li[Al(hfip)_4]$ (blue) (**1b**) in EC:EMC (3:7 v/v) solvent.

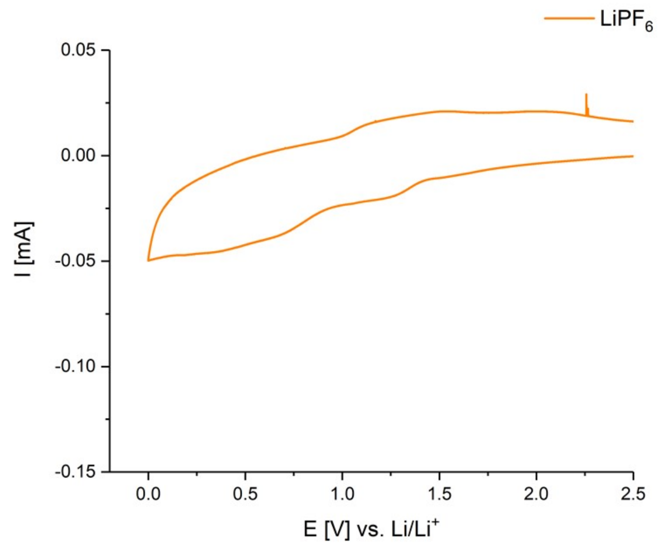

Figure S4.2.2 Cyclic voltammetry using a two-electrode cell, copper working electrode and lithium metal counter electrode. First cycle measured at a scan rate of 1 mV/s. Electrolyte is 1 M $LiPF_6$ in EC:EMC (3:7 v/v) solvent.

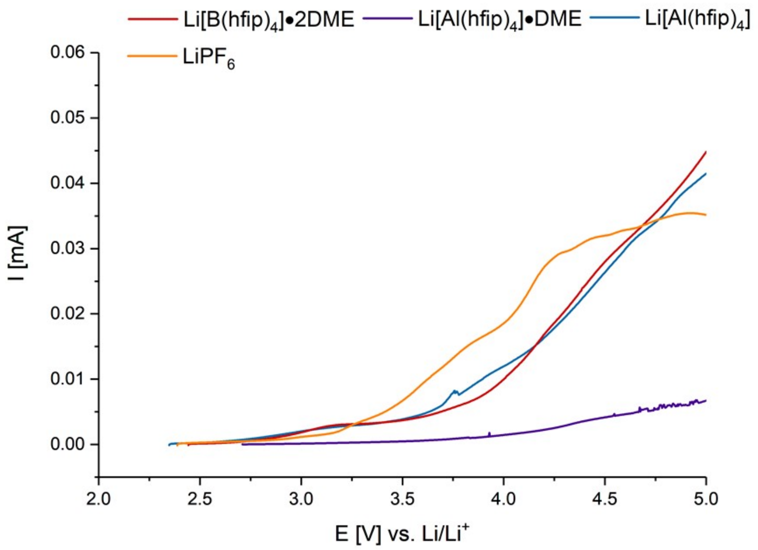

Figure S4.2.3 Cyclic voltammetry using a two-electrode cell, copper working electrode and lithium metal counter electrode. First cycle measured at a scan rate of 1 mV/s. Electrolytes are 1 M $Li[B(hfip)_4]\cdot 2DME$ (red) (**1a**·2DME), 1 M $Li[Al(hfip)_4]\cdot DME$ (purple) (**1b**·DME), 1 M $Li[Al(hfip)_4]$ (blue) (**1b**) and 1 M $LiPF_6$ (orange) in EC:EMC (3:7 v/v) solvent.

S4.3 Electrochemical impedance spectroscopy.

Galvanostatic electrochemical impedance spectroscopy (GEIS) was measured for the Cu||Li cells to assess the impedance of the electrochemical processes during plating. GEIS measurements with at −1.0 mA with excitation amplitude of 0.1 mA in the frequency range of 100 kHz to 0.1 Hz were performed. Plating current of −1.0 mA was applied for 1 minute before the GEIS measurement to ensure voltage stability. Kramers-Kronig compatibility test and equivalent circuit fits were performed using Gamry Ehem Analyst software. The equivalent circuit model consisted of solution resistance connected on series to two parallelly connected charge transfer resistance and constant phase element R(RQ)(RQ).

	Rs	$R_{Li\text{-}SEI}$	$Y_{Li\text{-}SEI}$	$a_{Li\text{-}SEI}$	$R_{Li\text{-}CT}$	$Y_{Li\text{-}CT}$	$a_{Li\text{-}CT}$
Li[B(hfip)$_4$]·2DME (**1a**·2DME)	6.14 ± 0.10	52.82 ± 0.50	3.6x10^{-6} ± 5.3 x10^{-7}	0.81	35.57 ± 2.00	5.20 x10^{-4} ± 3.2 x10^{-5}	0.7
Li[Al(hfip)$_4$]·DME (**1b**·DME)	5.62 ± 0.10	34.84 ± 0.42	6.8 x10^{-6} ± 1.2 x10^{-7}	0.71	22.27 ± 2.33	14.44 x10^{-4} ± 15.6 x10^{-5}	0.7
Li[Al(hfip)$_4$] (**1b**)	13.43 ± 0.23	35.51 ± 0.54	4.1 x10^{-6} ± 1.2 x10^{-7}	0.77	16.18 ± 1.24	11.81 x10^{-4} ± 18.2 x10^{-5}	0.7
LP57	5.00 ± 0.10	26.82 ± 0.32	6.8 x10^{-6} ± 1.5 x10^{-7}	0.70	19.15 ± 0.88	9.16 x10^{-4} ± 7.5 x10^{-5}	0.7

Table S4.3.1 Fitted equivalent circuit model parameters for the EIS data.

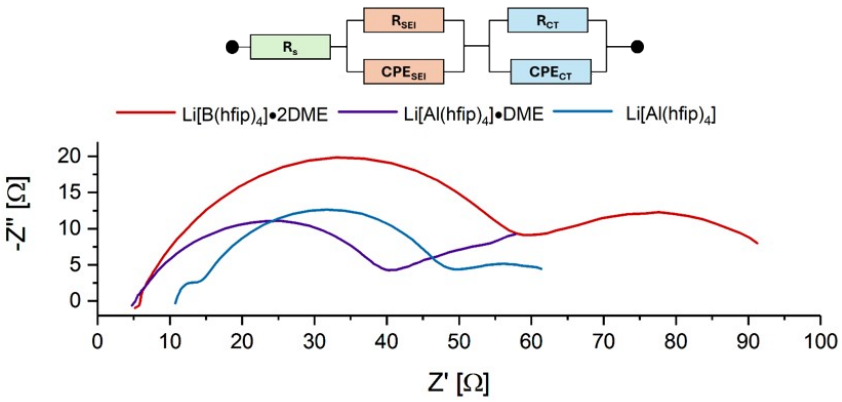

Figure S4.3.1 GEIS during plating of Cu||Li coin cell for 1 M Li[B(hfip)$_4$]·2DME (**1a**·2DME), 1 M Li[Al(hfip)$_4$]·DME (**1b**·DME) and 1 M Li[Al(hfip)$_4$] (**1b**) in EC:EMC (3:7 v/v) in the frequency range of 100 kHz to 0.1 Hz.

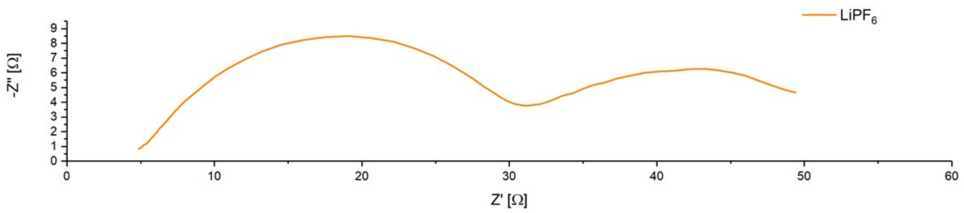

Figure S4.3.2 GEIS during plating of Cu||Li coin cell for 1 M $LiPF_6$ EC:EMC (3:7 v/v) (LP57) in the frequency range of 100 kHz to 0.1 Hz.

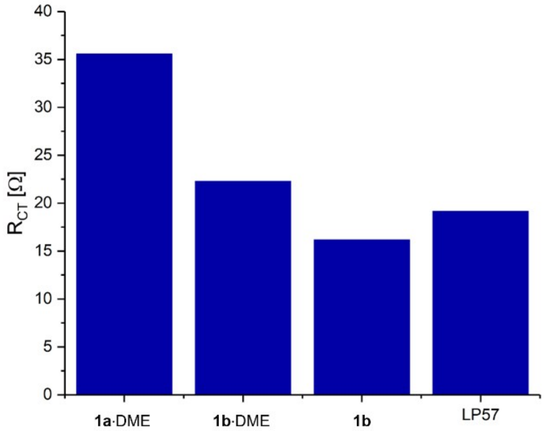

Figure S4.3.3 Charge transfer resistance determined by EIS for 1 M Li[B(hfip)$_4$]·2DME (**1a**·2DME), 1 M Li[Al(hfip)$_4$]·DME (**1b**·DME) and 1 M Li[Al(hfip)$_4$] (**1b**) in EC:EMC (3:7 v/v) in the frequency range of 100 kHz to 0.1 Hz.

S4.4 Lithium metal cycling

Galvanostatic cycling was performed on Biologic VSP potentiostat/galvanostat with Cu||Li cell containing 1 M of the prepared electrolytes. The copper foil was cut to 12.7 mm diameter (1.27 cm^2 area) and 0.5 mA currents were applied for 30 minutes with ±2.5V *vs*. Li/Li^+ voltage limits. This current density was 0.4 mA cm^{-2} and aerial capacity was 0.2 mAh cm^{-2}.

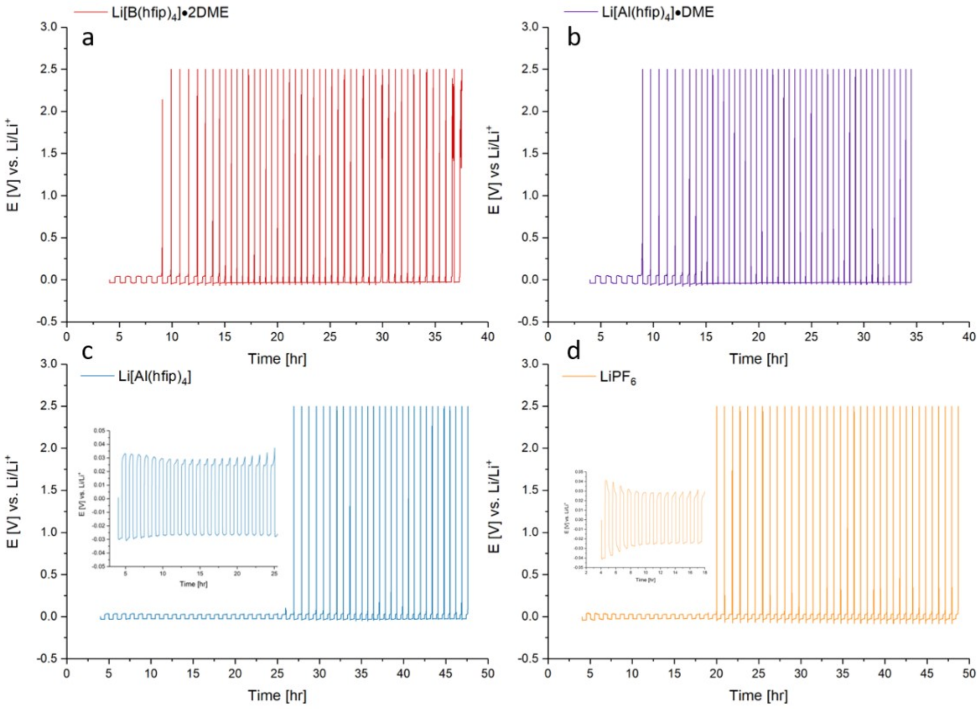

Figure S4.4.1 Lithium vs. copper cell cycling using 1 M Li[B(hfip)$_4$]·2DME (**1a**·2DME), 1 M Li[Al(hfip)$_4$]·DME (**1b**·DME), 1 M Li[Al(hfip)$_4$] (**1b**) and 1 M $LiPF_6$ in EC:EMC (3:7 v/v) as electrolytes. Current density 0.4 mA cm^{-2} and aerial capacity 0.2 mAh cm^{-2}.

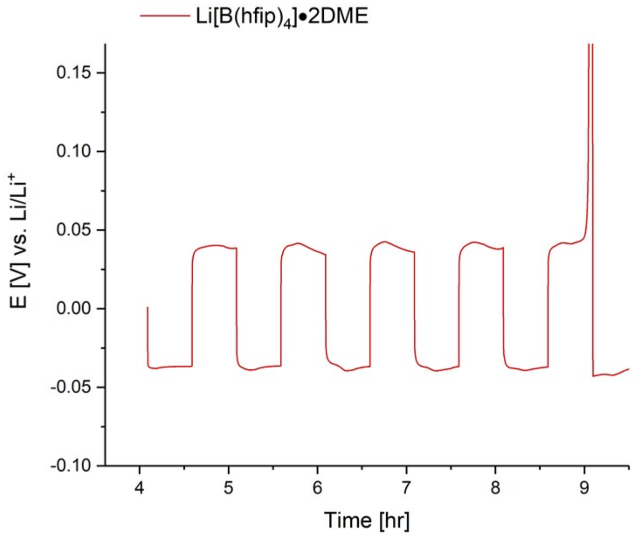

Figure S4.4.2 Lithium vs. copper cell cycling using 1 M Li[B(hfip)$_4$]·2DME (**1a**·2DME) in EC:EMC (3:7 v/v) as electrolyte.

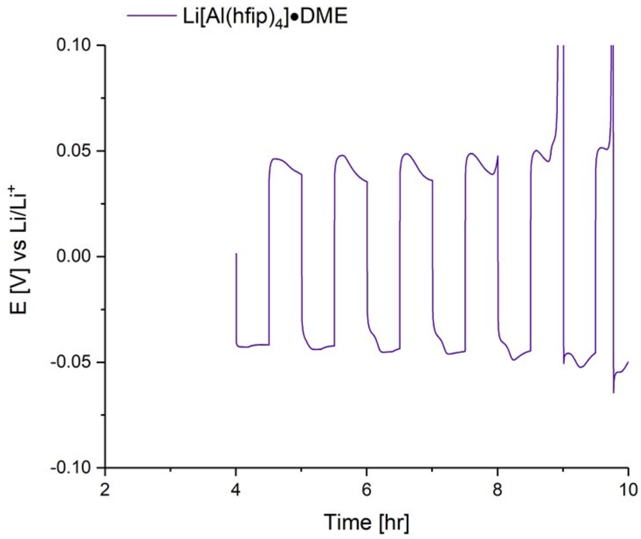

Figure S4.4.3 Lithium vs. copper cell cycling using 1 M Li[Al(hfip)$_4$]·DME (**1b**·DME) in EC:EMC (3:7 v/v) as electrolyte.

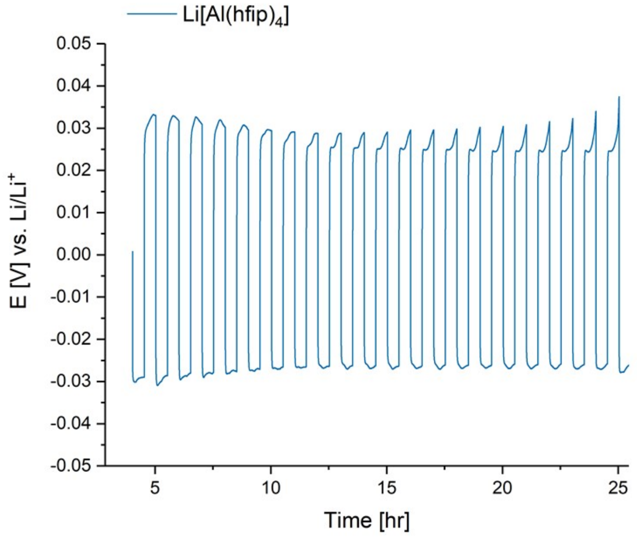

Figure S4.4.4 Lithium vs. copper cell cycling using 1 M Li[Al(hfip)$_4$] (**1b**) in EC:EMC (3:7 v/v) as electrolyte.

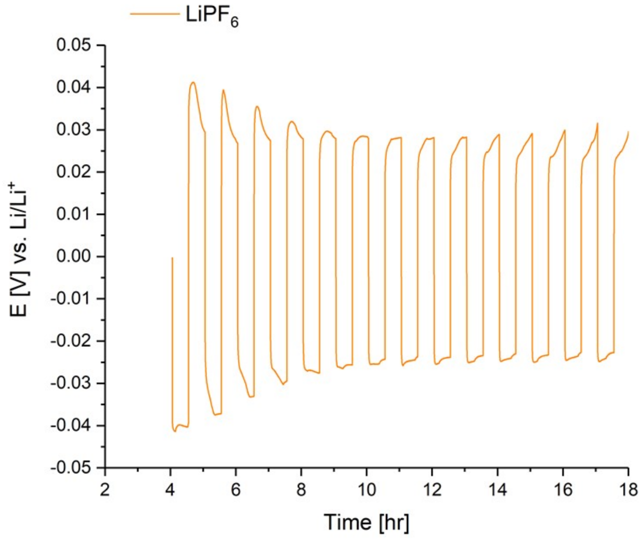

Figure S4.4.5 Lithium vs. copper cell cycling using 1 M $LiPF_6$ in EC:EMC (3:7 v/v) (LP57) as electrolyte.

S4.5 Lithium-ion cycling.

Coin cells (2032 from Cambridge energy solutions) were prepared in an argon glovebox (O2 < 1 ppm, H2O < 1 ppm). For Li-ion battery cells, 2-electrode single-layer coin cells with 1.77 cm^2 cathode area were assembled with a geometrically over-sized hard carbon anode (2.01 cm^2). $LiNi_{0.8}Mn_{0.1}Co_{0.1}O_2$ (NMC811) and graphite printed electrode foils were provided by the Cell Analysis, Modeling and Prototype (CAMP) facility at Argonne National Lab (USA). The NMC cathode (batch code A-C020) consisted of 90 wt% NMC811 (Targray), 5 wt% PVDF binder (Solvay 5130) and 5 wt% carbon black (Timcal C45) coated onto an aluminium current collector. This had a mass loading of active material of 8.3 mg cm^{-2} and practical capacity of 195 $mAhg^{-1}$. The graphite electrode consisted of 91.83 wt% graphite powder (Hitachi MagE3), 2 wt% carbon black (Timcal C45), 6 wt% PVDF binder (Kureha 9300) and 0.17 wt% oxalic acid coated onto a copper current collector. This had a mass loading of 5.8 mg cm^{-2} and a practical capacity of 360 $mAhg^{-1}$.[3,4]

Li-ion coin cells were tested at room-temperature with a battery cycler (MPG 200, Biologic Instruments). The formation protocol consisted of 2 charge-discharge cycles at a rate of C/3 between a voltage limit of 4.2–2.5 V. For cycle life tests, a 1C charge/discharge (constant current- constant voltage, CCCV profile) rate was used. The C-rates was based on a nominal specific capacity of 195 $mAhg^{-1}$ for the cathode. Glass fibre, which was dried under vacuum (1×10^{-2} mbar) at 100 °C for 18 hours, was used as the separator, with 100 µL of electrolyte.

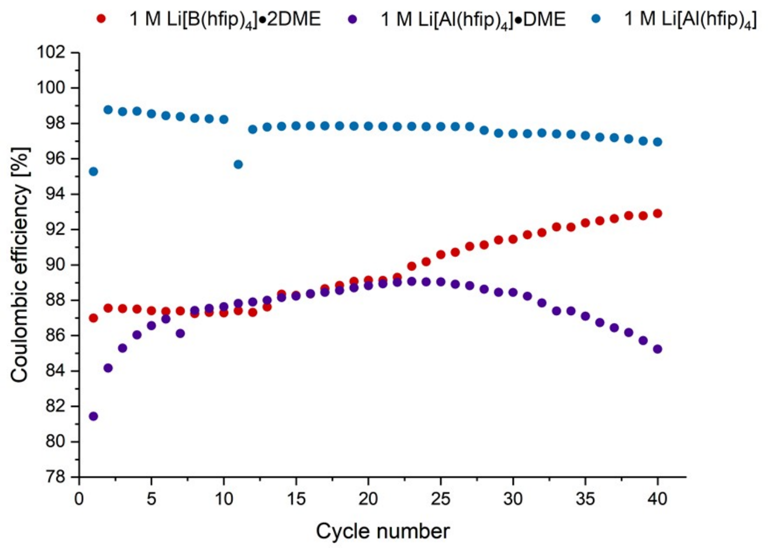

Figure S4.5.1 Coulombic efficiency vs. cycle number for 1 M $Li[B(hfip)_4]\cdot 2DME$ (**1a**·2DME) (red), 1 M $Li[Al(hfip)_4]\cdot DME$ (**1b**·DME) (purple) and 1 M $Li[Al(hfip)_4]$ (**1b**) (blue) in EC:EMC electrolytes. Approximate constant current rate of 1C for charge and discharge using cell voltage limits of 4.2 and 2.5 V.

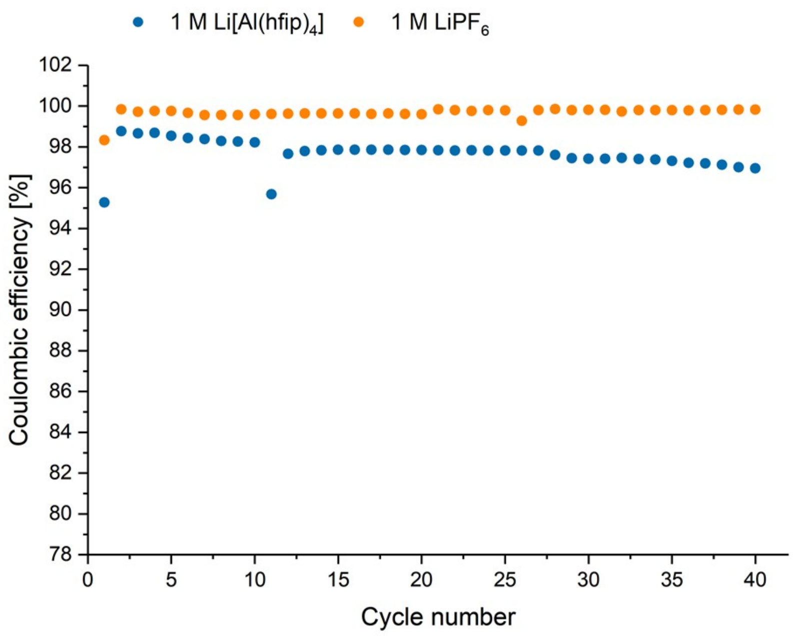

Figure S4.5.2 Coulombic efficiency vs. cycle number for 1 M Li[Al(hfip)$_4$] (**1b**) (blue) in EC:EMC and 1 M LiPF$_6$ in EC:EMC (LP57, orange) electrolytes. Approximate constant current rate of 1C for charge and discharge using cell voltage limits of 4.2 and 2.5 V.

S4.5 Electrolyte preparation.

Electrolyte solutions were prepared by dissolving the appropriate amount of the lithium salt Li[B(hfip)$_4$]·2DME (red) (**1a**·2DME), Li[Al(hfip)$_4$]·DME (purple) (**1b**·DME) or Li[Al(hfip)$_4$] (blue) (**1b**) in EC:EMC (3:7 v/v) solvent to make a 1 M concentration. Electrolytes were prepared and stored in aluminium bottles in an argon filled glovebox. Air sensitive techniques and drying the salts at 85–90 °C under vacuum (1 × 10^{-2} mbar), as well as drying the EC:EMC solvent over activated 4 Å molecular sieves before use, was performed to minimise the water content of the electrolyte solutions.

For lithium tetrakis(hexafluoroisopropoxy)borate, it was decided to use the 2 DME solvating molecules as found by solution-state ^{1}H NMR spectroscopy to give 1 M Li[B(hfip)$_4$]·2DME in EC:EMC. However, if the structure had 1 DME molecule, the difference in molecular weight between Li[B(hfip)$_4$]·2DME (Mr = 866.12) and Li[B(hfip)$_4$]·DME (Mr = 775.99) when preparing the electrolyte using the molecular weight of 2 solvating DME molecules would mean that the electrolyte concentration would have been 1.1 M.

S5 Viscosity measurements.

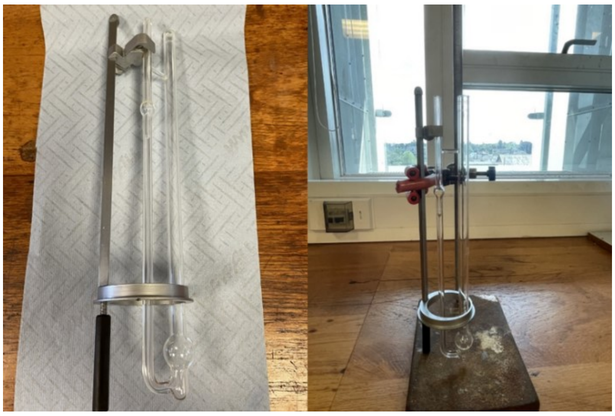

Figure S5.1: Photographs showing the Micro-Ostwald viscometer using to determine the viscosity of Li[B(hfip)$_4$]·2DME (**1a**·2DME), Li[Al(hfip)$_4$]·DME (**1b**·DME) and Li[Al(hfip)$_4$] (**1b**) .

Kinematic viscosities were measured in an argon glovebox with a Micro-Ostwald viscometer, type 51620/II (SI Analytics), with an instrument constant K = 0.1063 mm^2 s^{-2}. 2 ml of the liquid was filled into the viscometer, and the time taken to fall was measured four times and averaged. The temperature inside the glovebox was approximately 28 °C. Kinematic viscosities were converted to dynamic viscosities by multiplying by liquid density; density was measured by weighing 2 ml of liquid in an argon glovebox. The density of Li[B(hfip)$_4$]·2DME (**1a**·2DME) = 1.38 g/ml, Li[Al(hfip)$_4$]·DME (**1b**·DME) = 1.26 g/ml, Li[Al(hfip)$_4$] (**1b**) = 1.28 g/ml and LP57 = 1.23 g/ml.

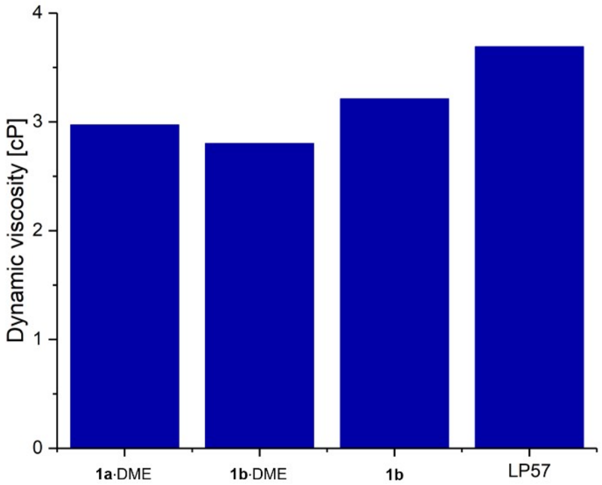

Figure S5.2: Dynamic viscosity of electrolyte solutions 1 M Li[B(hfip)$_4$]·2DME (**1a**·2DME), Li[Al(hfip)$_4$]·DME (**1b**·DME) and Li[Al(hfip)$_4$] (**1b**) in EC:EMC (3:7 v/v) solvent, measured at 28 °C.

S6 NMR measurements.

S6.1 Solid-state NMR spectroscopy measurements.

^{1}H, ^{27}Al and ^{19}F MAS experiments were acquired on a 700 MHz (16.4 T) magnet equipped with Avance IIIHD console using a Bruker 1.3mm HXY probe. The ^{1}H, ^{19}F and ^{27}Al spectra were recorded at a MAS frequency of 40 kHz, ran with a recycle delays of 4s, 2s and 2s and reference to H_2O, LiF and AlF_3, respectively. A one pulse experiment was used for the ^{1}H and ^{27}Al spectra, whilst an aring sequence was used to record the^{19}F spectrum to suppress probe background.

S6.2 Solution NMR spectroscopy measurements.

Solution NMR experiments were conducted on a Bruker 11.7 T magnet ($\nu_0(^{1}H)$ = 500.20 MHz, $\nu_0(^{19}F)$ = 470.62 MHz) equipped with an Avance IIIHD console. A 5 mm BBO probe was used for all experiments. Diffusion coefficients were measured using a bipolar pulsed gradient stimulated echo experiment with spoil gradients. Diffusion times were 10 ms and gradient pulse lengths of 1.2 ms and 2.5 ms were used for ^{1}H and ^{19}F, respectively. The gradient strengths were varied and the diffusion coefficients were determined by fitting the signal decay using the Stejskal-Tanner equation.

Table S6.2 Diffusion coefficients for the various ^{1}H environments in the electrolyte solvent (EC:EMC 3:7 v/v) and the ^{19}F in the anions. All values are in units of $10^{-10}m^2/s$.

Compound	EC	EMC -CH_2-	EMC -OMe	EMC -CH_3	DME	DME	^{19}F
Li[Al(hfip)$_4$]	3.97	4.87	4.80	5.05	-	-	2.15
Li[Al(hfip)$_4$]•DME	4.84	6.16	6.13	5.85	4.26	4.46	2.13
Li[B(hfip)$_4$]•2DME	5.59	7.07	7.37	5.39	5.45	7.08	2.24
LP57	4.17	3.89	3.95	5.05	-	-	2.09

S7 References.

1 B. Roy, P. Cherepanov, C. Nguyen, C. Forsyth, U. Pal, T. C. Mendes, P. Howlett, M. Forsyth, D. MacFarlane and M. Kar, *Adv. Energy Mater.*, 2021, **11**, 2101422.

2 S. M. Ivanova, B. G. Nolan, Y. Kobayashi, S. M. Miller, O. P. Anderson and S. H. Strauss, *Chem. Eur. J.*, 2001, **7**, 503–510.

3 M.-T. F. Rodrigues, K. Kalaga, S. E. Trask, I. A. Shkrob and D. P. Abraham, *J. Electrochem. Soc.*, 2018, **165**, A1697–A1705.

4 Z. Ruff, C. Xu and C. P. Grey, *J. Electrochem. Soc.*, 2021, **168**, 060518.

S8 NMR spectra.

S8.1 NMR spectra of lithium borate and aluminate complexes

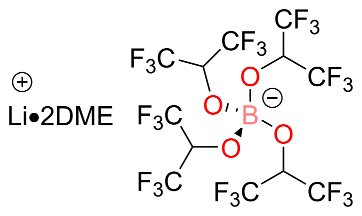

Figure S8.1.1 ^{1}H NMR (400 MHz, CD_3CN, 295 K) spectrum of lithium tetrakis(hexafluoroisopropoxy)borate, Li[B(hfip)$_4$]·2DME (**1a**·2DME).

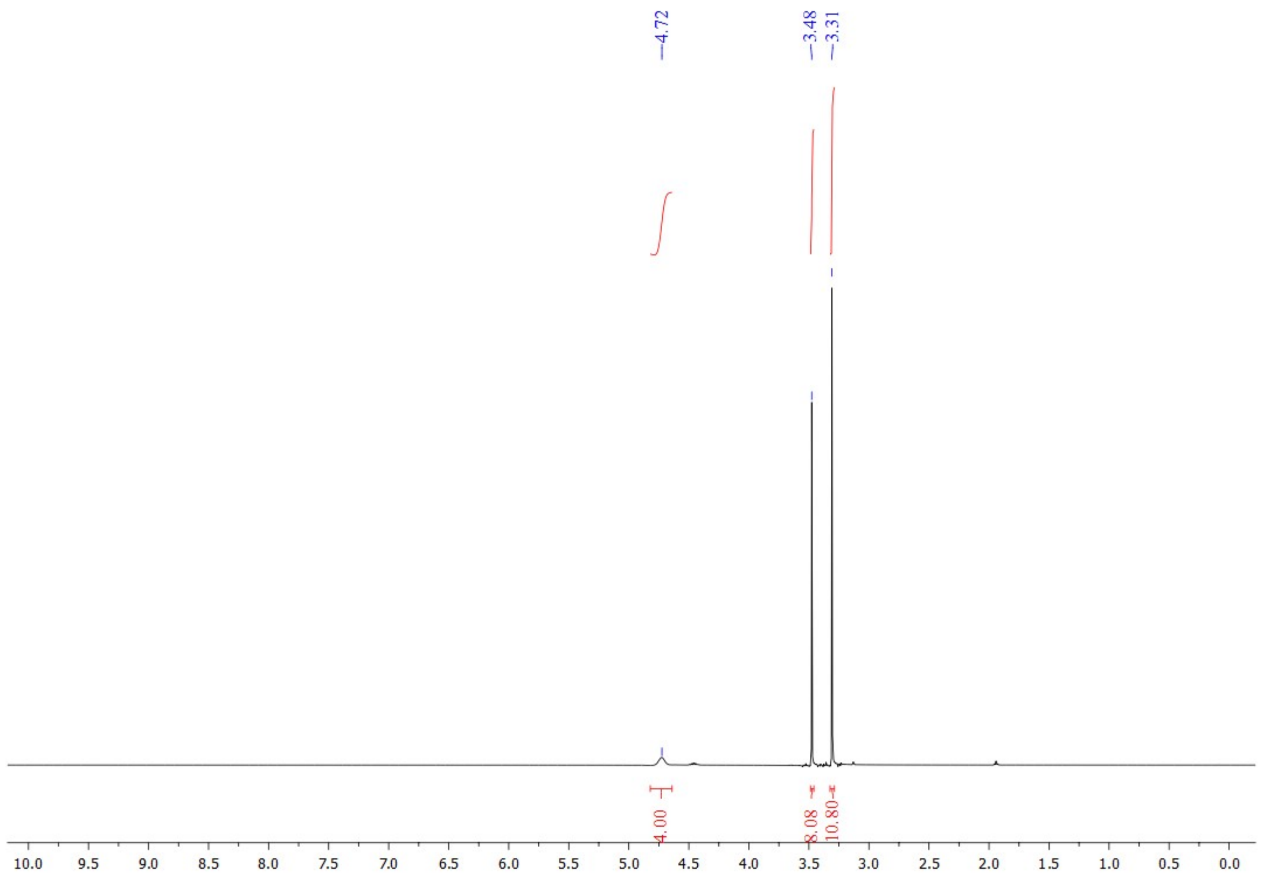

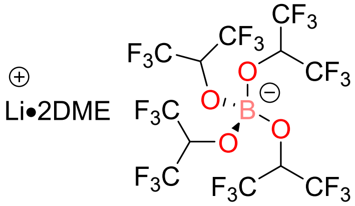

Figure S8.1.2 $^{13}C\{^{1}H\}$ NMR (101 MHz, $(CD_3)_2SO$, 295 K) spectrum of lithium tetrakis(hexafluoroisopropoxy)borate, $Li[B(hfip)_4]\cdot 2DME$ (**1a**·2DME).

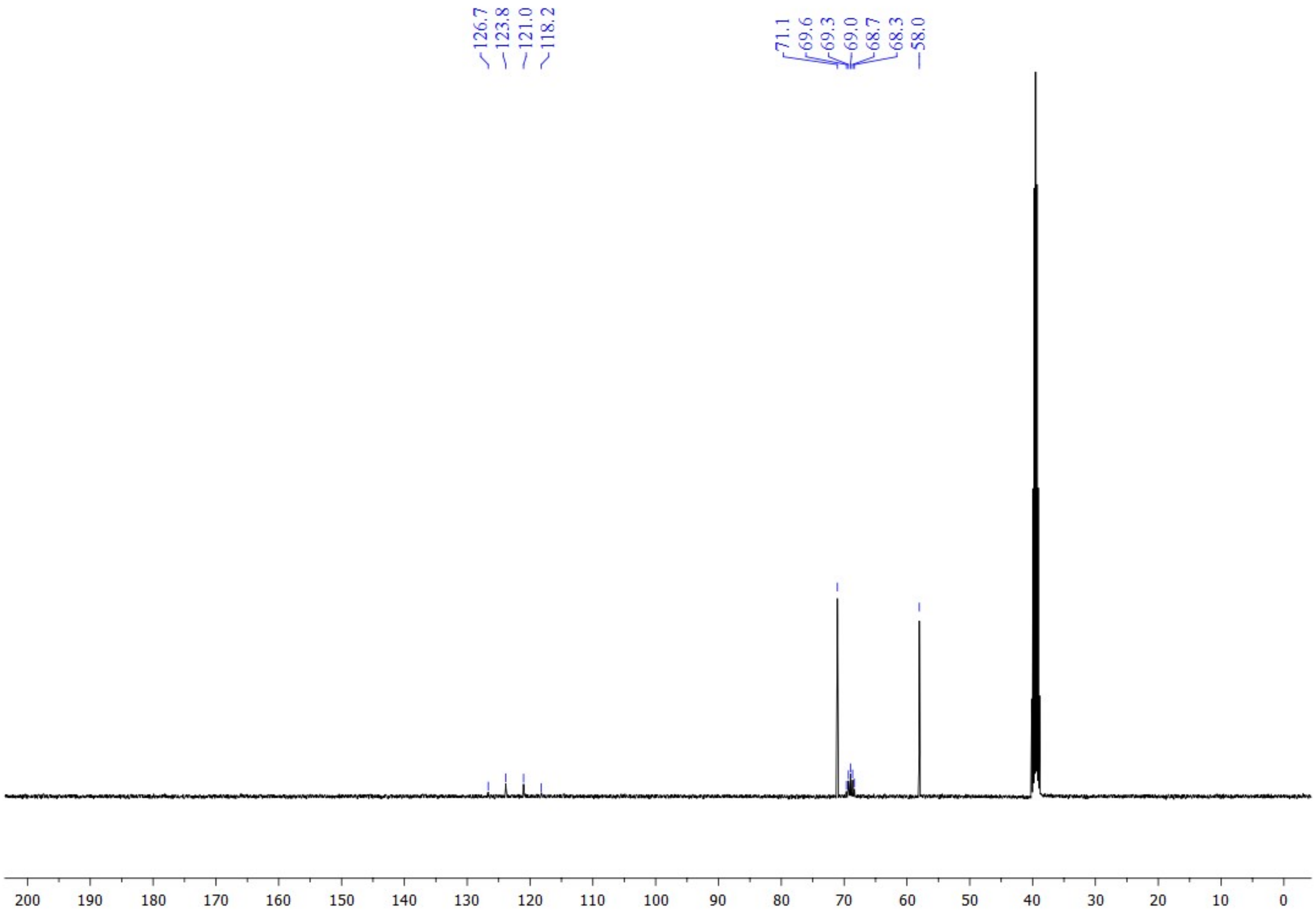

Figure S8.1.3 ^{11}B NMR (128 MHz, CD_3CN, 295 K) spectrum of lithium tetrakis(hexafluoroisopropoxy)borate, Li[B(hfip)$_4$]·2DME (**1a**·2DME).

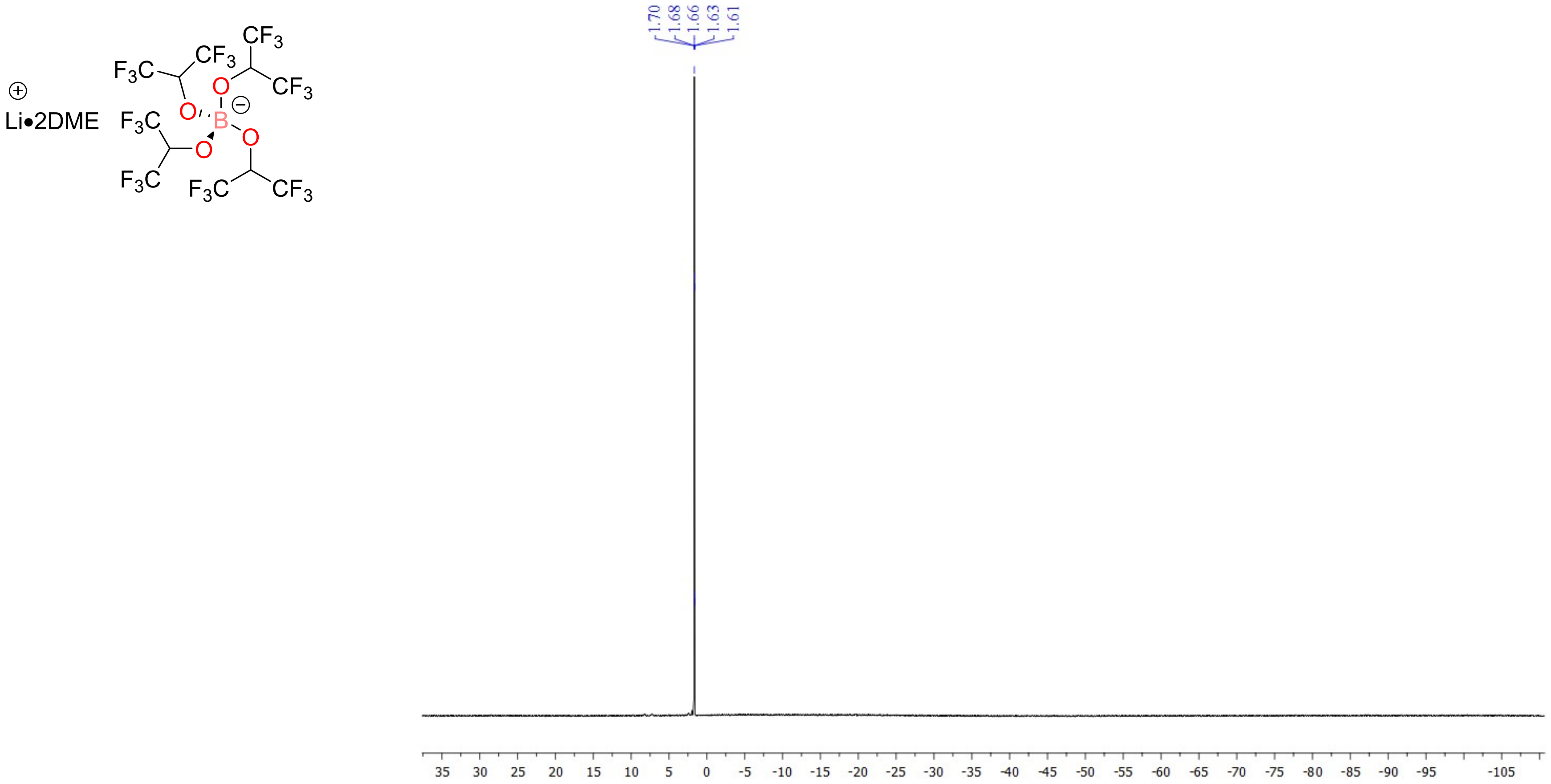

Figure S8.1.4 ^{19}F NMR (376 MHz, CD_3CN, 295 K) spectrum of lithium tetrakis(hexafluoroisopropoxy)borate, Li[B(hfip)$_4$]·2DME (**1a**·2DME).

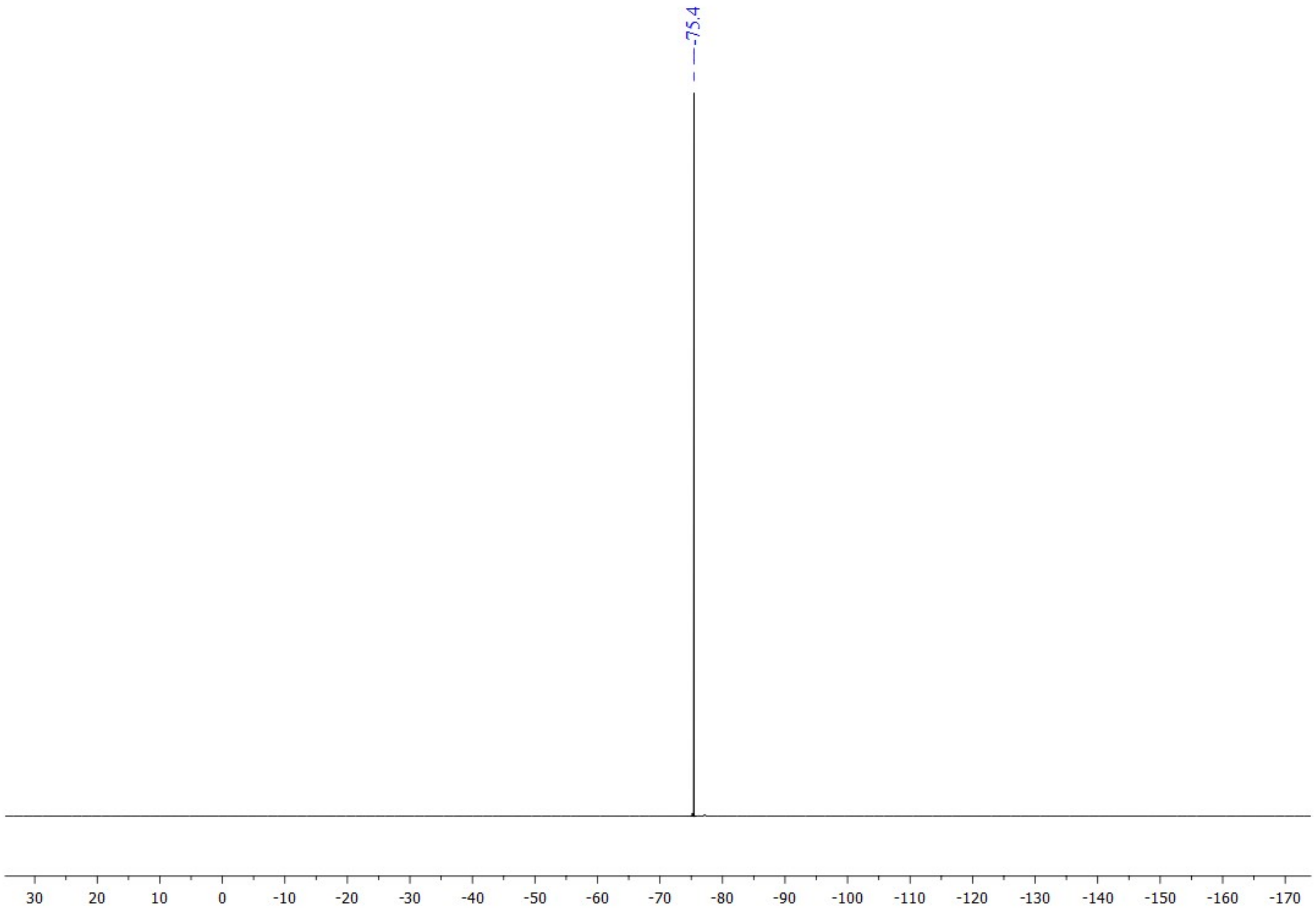

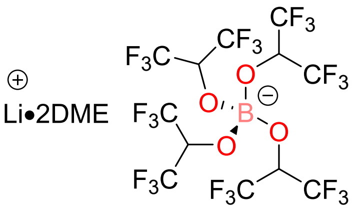

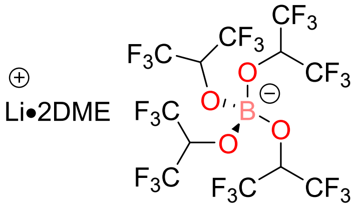

Figure S8.1.5 ^{7}Li NMR (155 MHz, $(CD_3)_2SO$, 295 K) spectrum of lithium tetrakis(hexafluoroisopropoxy)borate, $Li[B(hfip)_4]\cdot 2DME$ (**1a**·2DME).

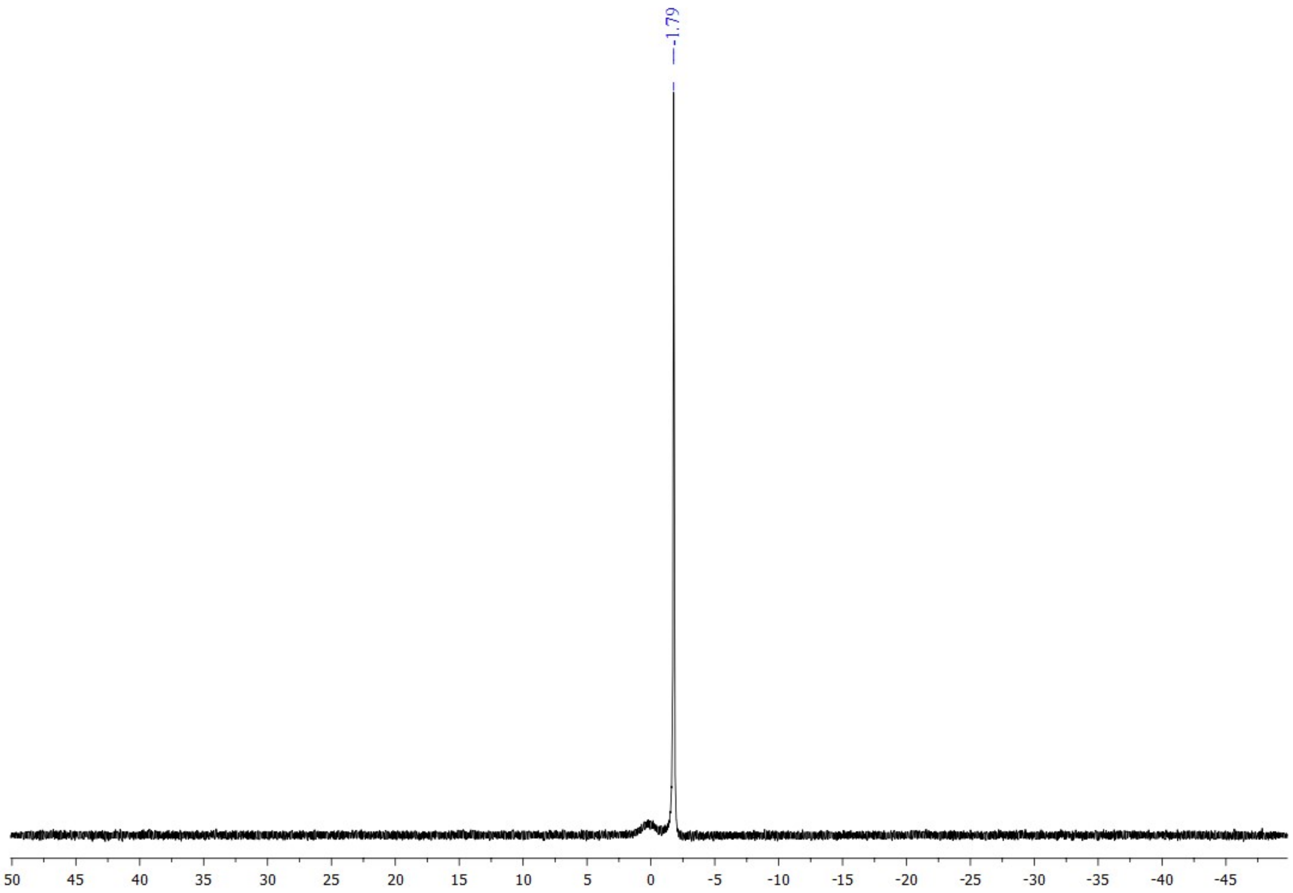

Figure S8.1.6 ^{1}H NMR (400 MHz, CD_3CN, 295 K) spectrum of lithium tetrakis(hexafluoroisopropoxy)aluminate, $Li[Al(hfip)_4]\cdot DME$ (**1b**·DME).

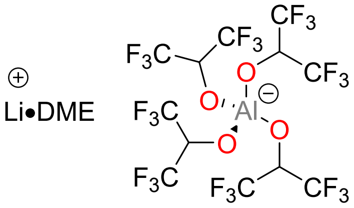

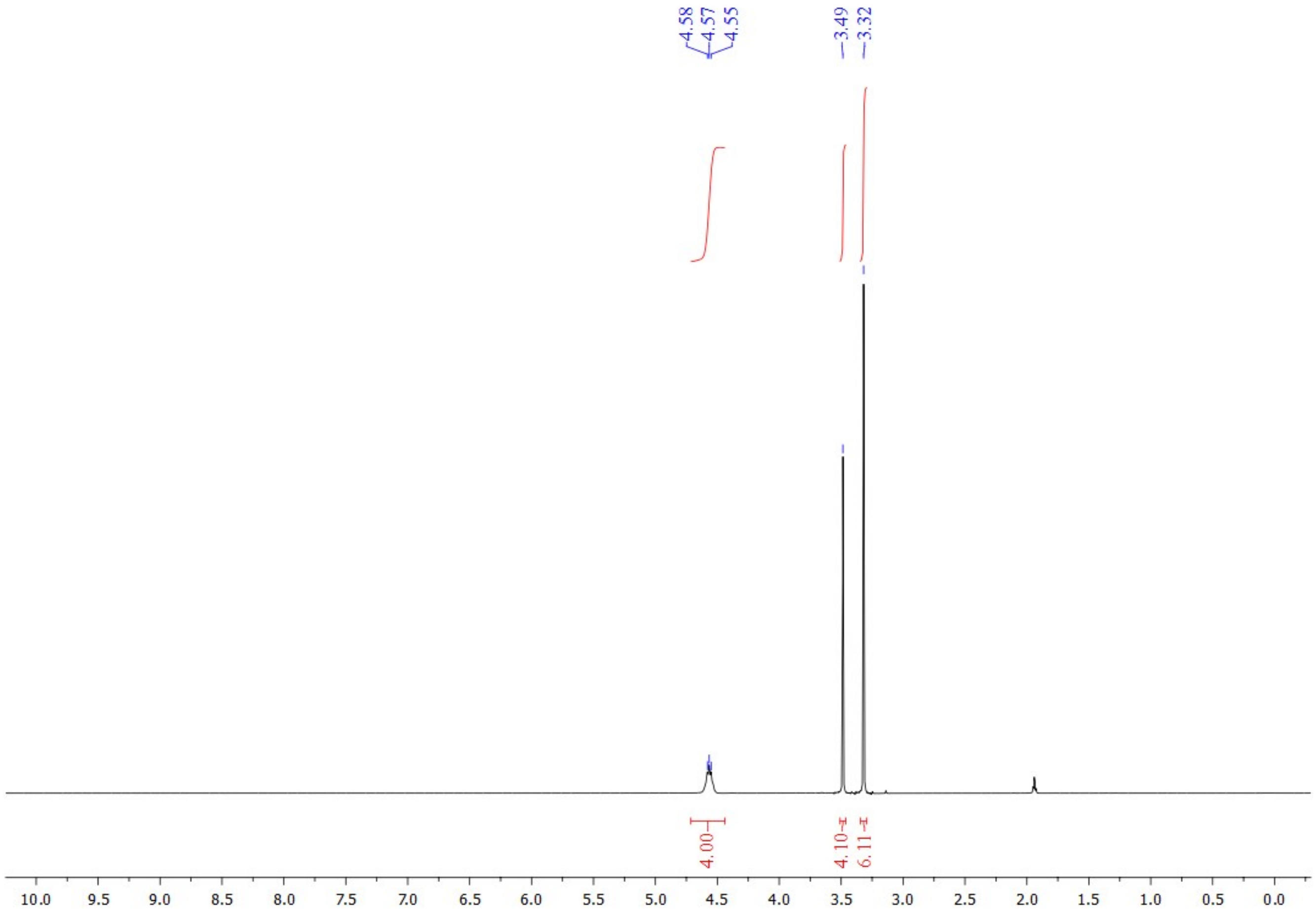

4.58
4.57
4.55
3.49
3.32
4.00
4.10
6.11
10.0 9.5 9.0 8.5 8.0 7.5 7.0 6.5 6.0 5.5 5.0 4.5 4.0 3.5 3.0 2.5 2.0 1.5 1.0 0.5 0.0

Figure S8.1.7 $^{13}C\{^1H\}$ NMR (101 MHz, CD_3CN, 295 K) spectrum of lithium tetrakis(hexafluoroisopropoxy)aluminate, $Li[Al(hfip)_4]\cdot DME$ (**1b**·DME).

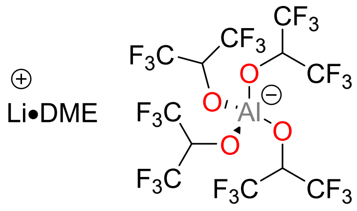

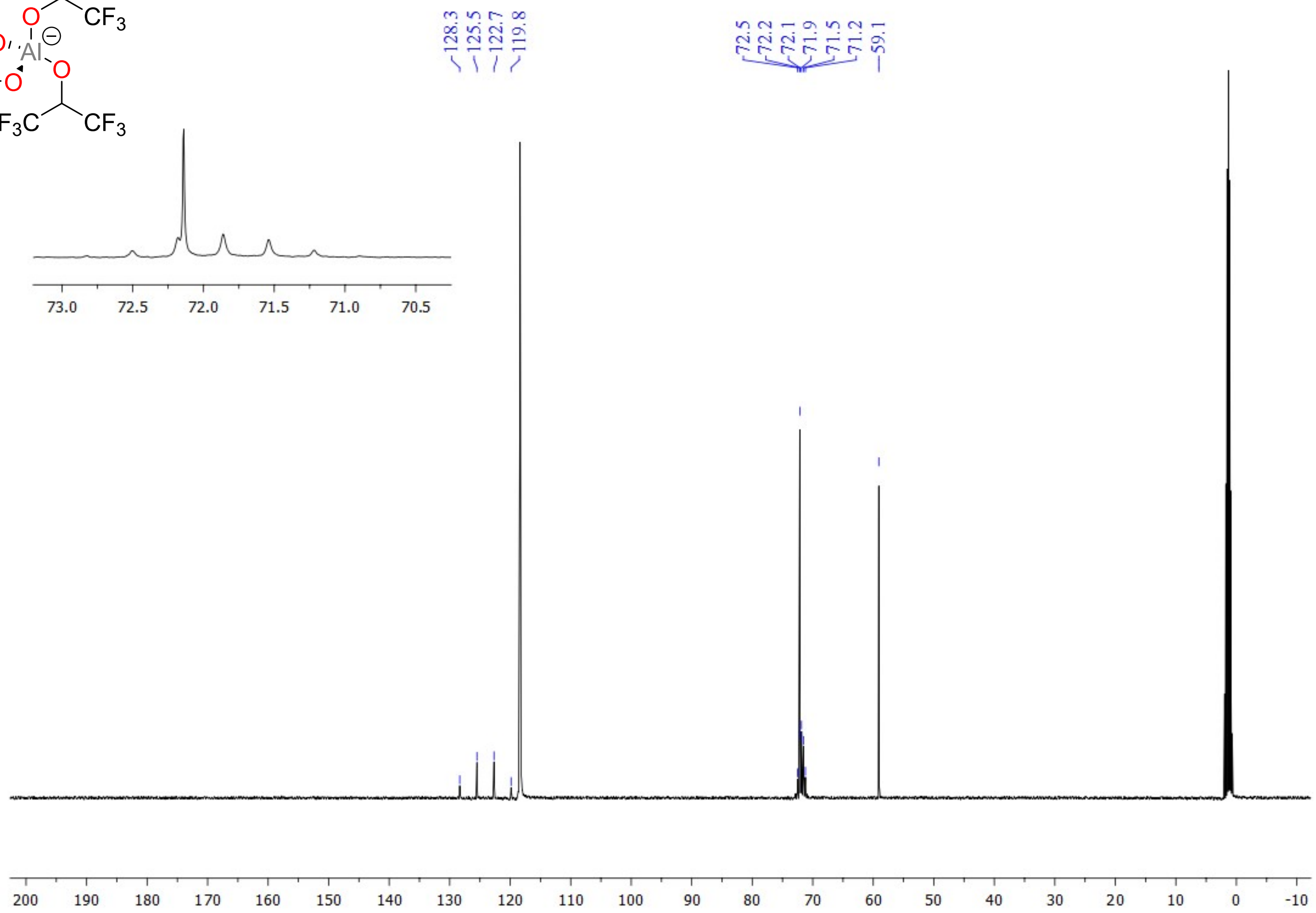

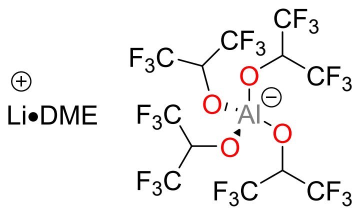

Figure S8.1.8 ^{27}Al NMR (104 MHz, CD_3CN, 295 K) spectrum of lithium tetrakis(hexafluoroisopropoxy)aluminate, Li[Al(hfip)$_4$]·DME (**1b**·DME).

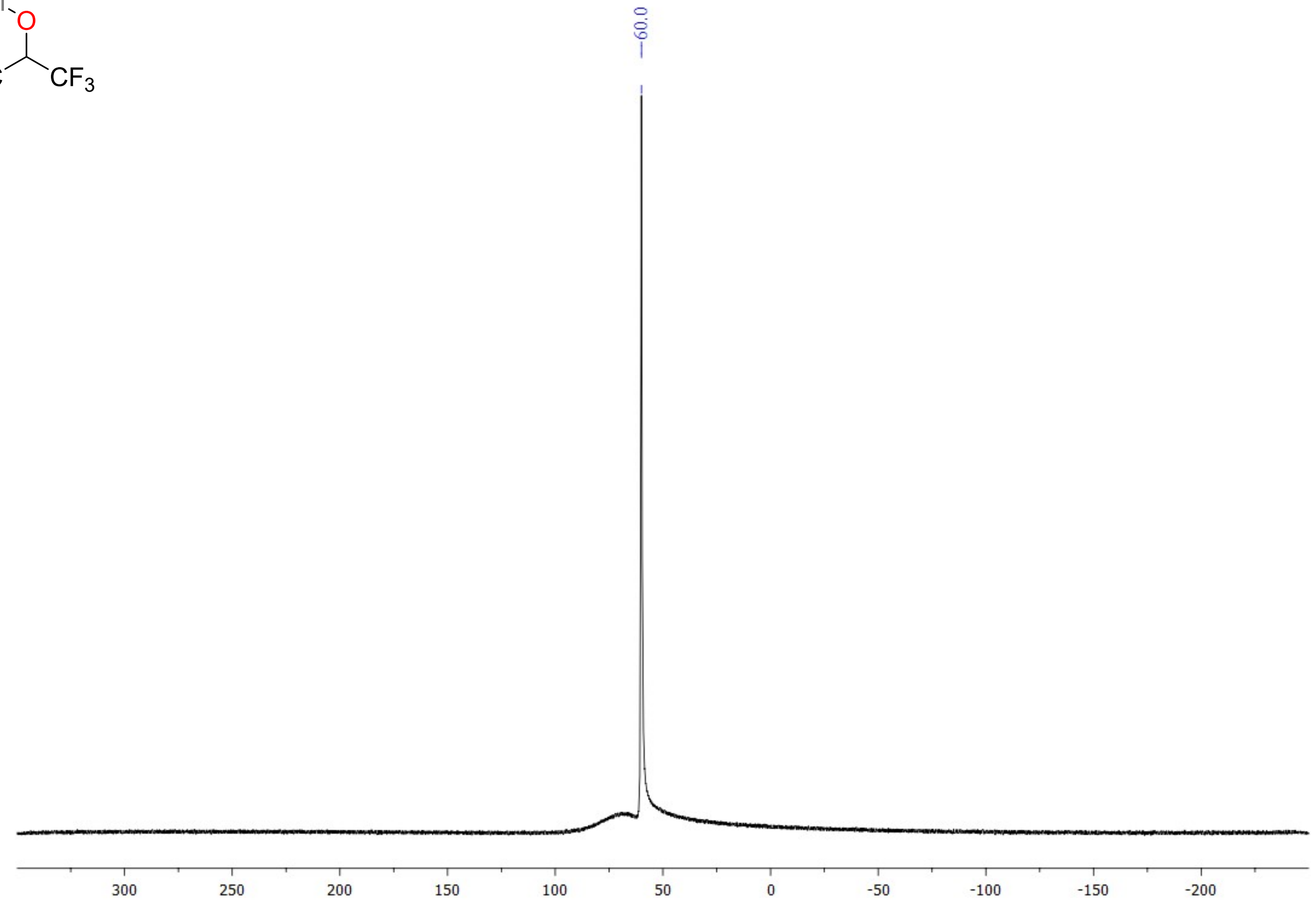

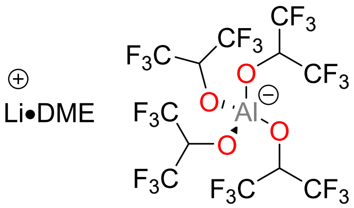

Figure S8.1.9 ^{19}F NMR (376 MHz, CD_3CN, 295 K) spectrum of lithium tetrakis(hexafluoroisopropoxy)aluminate, $Li[Al(hfip)_4]\cdot DME$ (**1b**·DME).

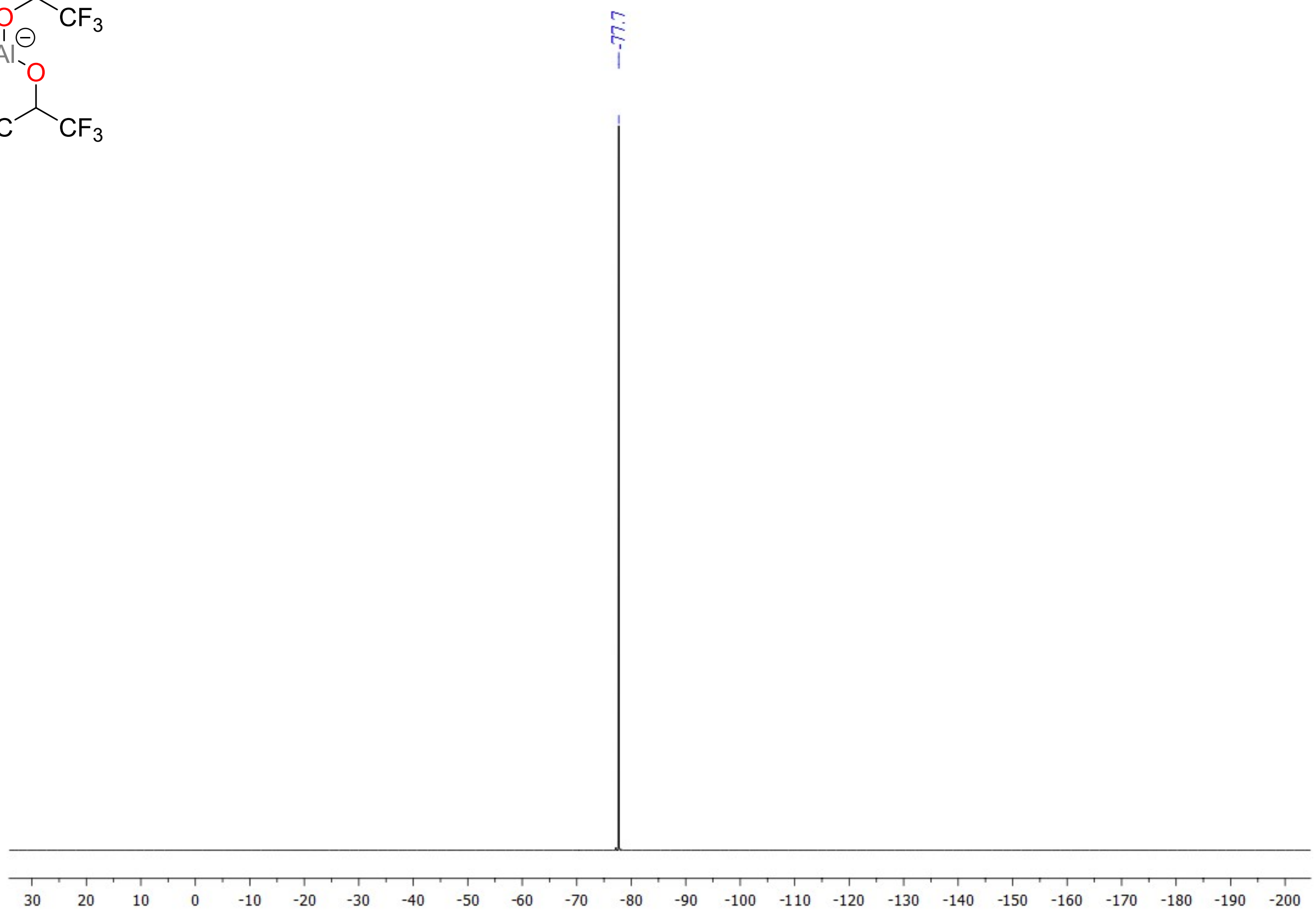

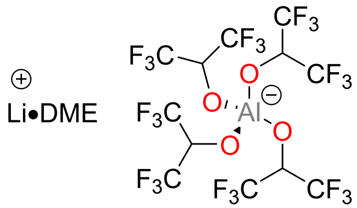

Figure S8.1.10 ^{7}Li NMR (155 MHz, CD_3CN, 295 K) spectrum of lithium tetrakis(hexafluoroisopropoxy)aluminate, Li[Al(hfip)$_4$]·DME (**1b**·DME).

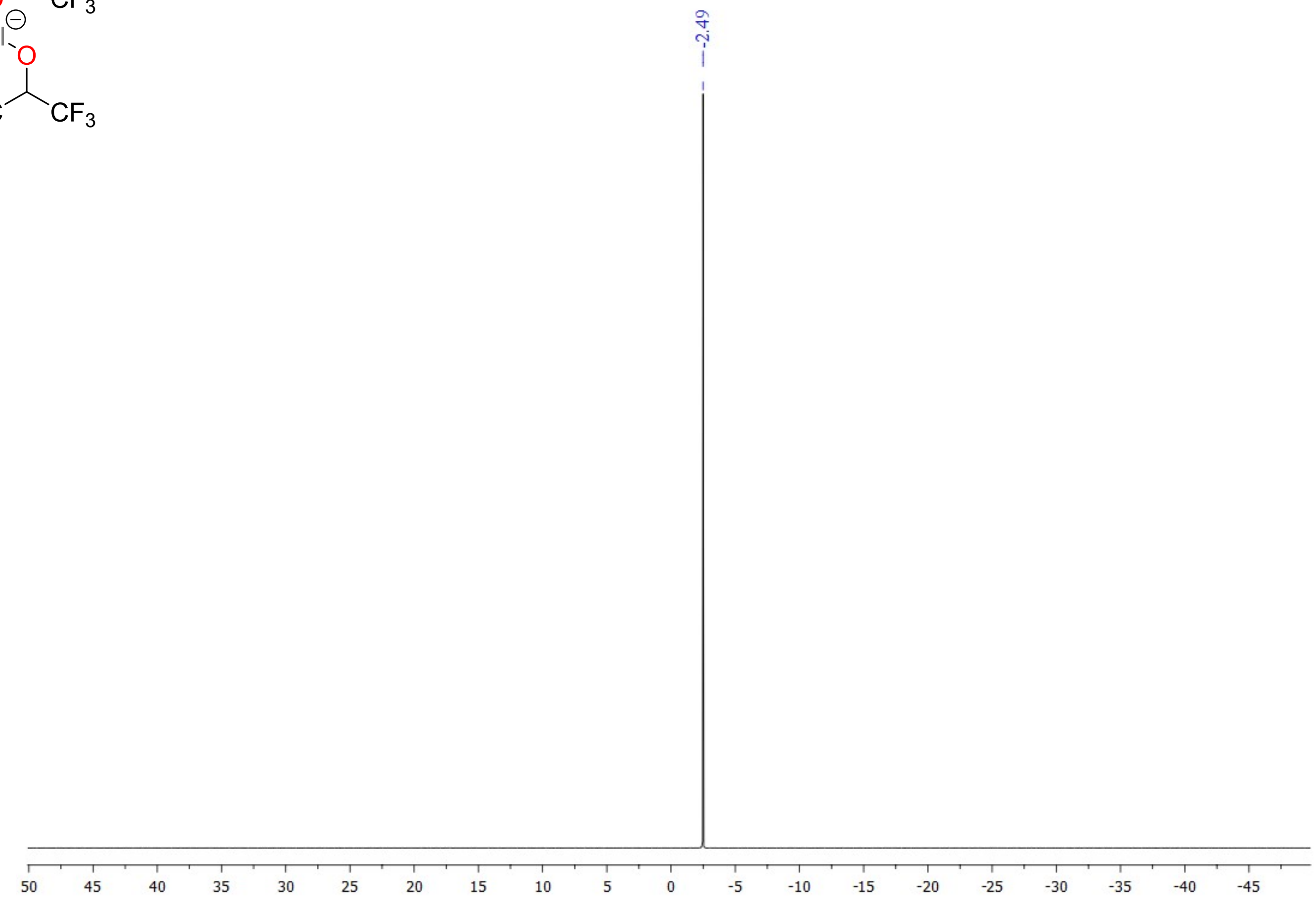

Figure S8.1.11 ^{1}H NMR (400 MHz, CD_3CN, 295 K) spectrum of lithium tetrakis(hexafluoroisopropoxy)aluminate, Li[Al(hfip)$_4$] (**1b**).

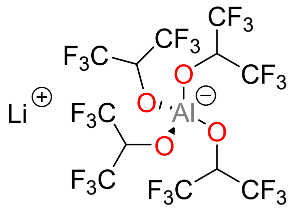

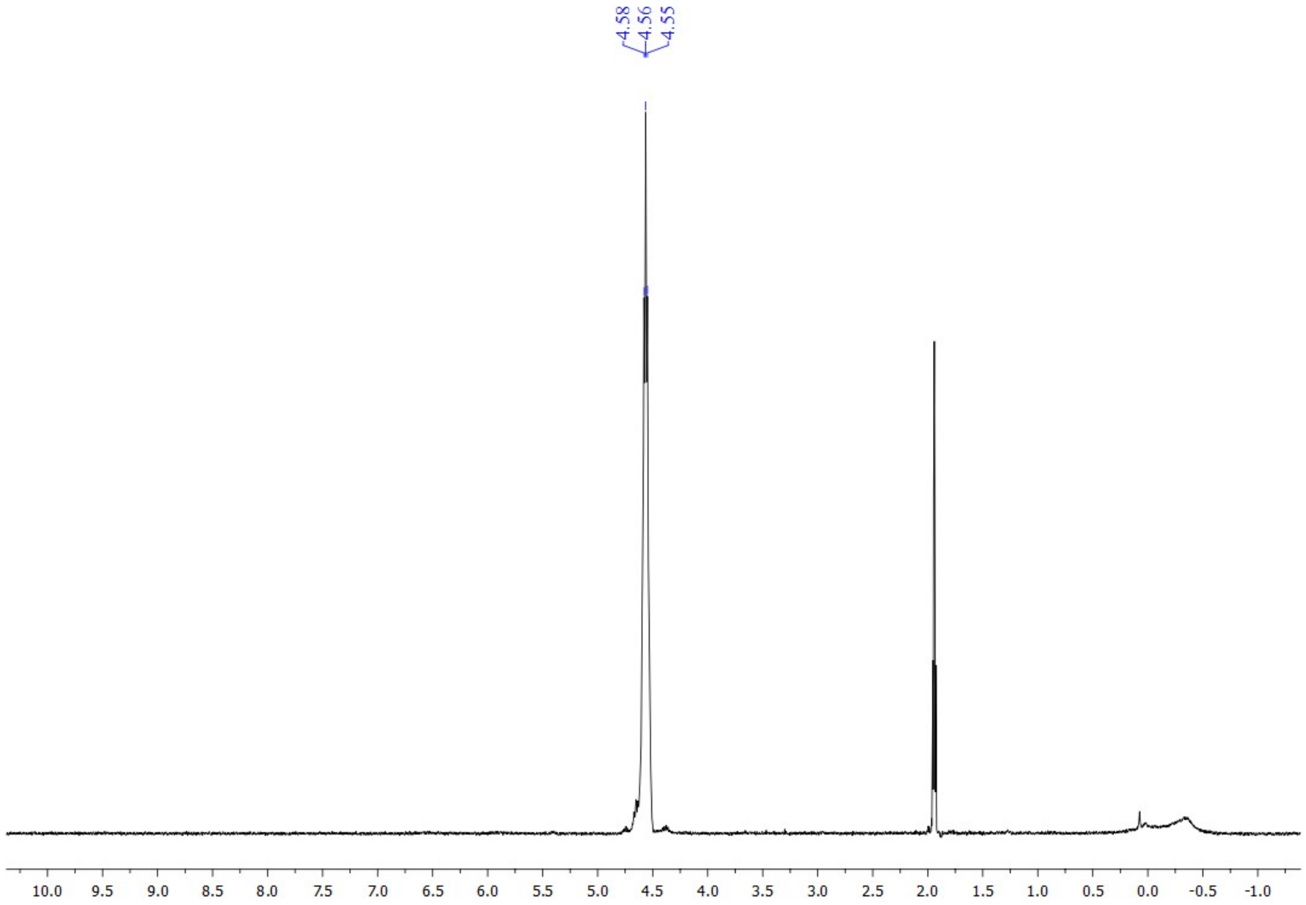

Figure S8.1.12 $^{13}C\{^1H\}$ NMR (101 MHz, CD_3CN, 295 K) spectrum of lithium tetrakis(hexafluoroisopropoxy)aluminate, Li[Al(hfip)$_4$] (**1b**).

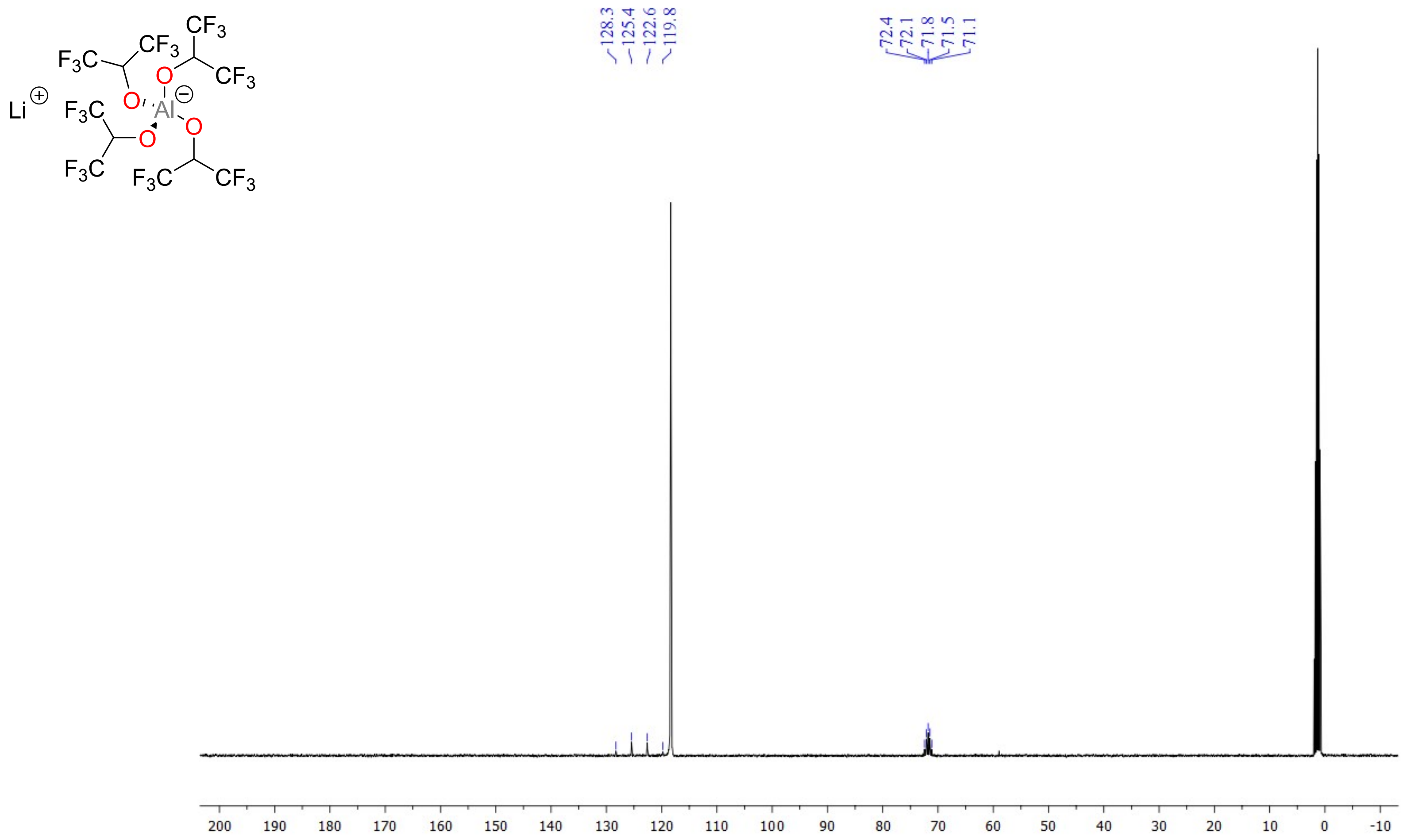

Figure S8.1.13 ^{27}Al NMR (104 MHz, CD_3CN, 295 K) spectrum of lithium tetrakis(hexafluoroisopropoxy)aluminate, Li[Al(hfip)$_4$] (**1b**).

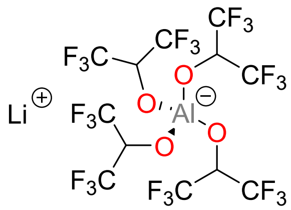

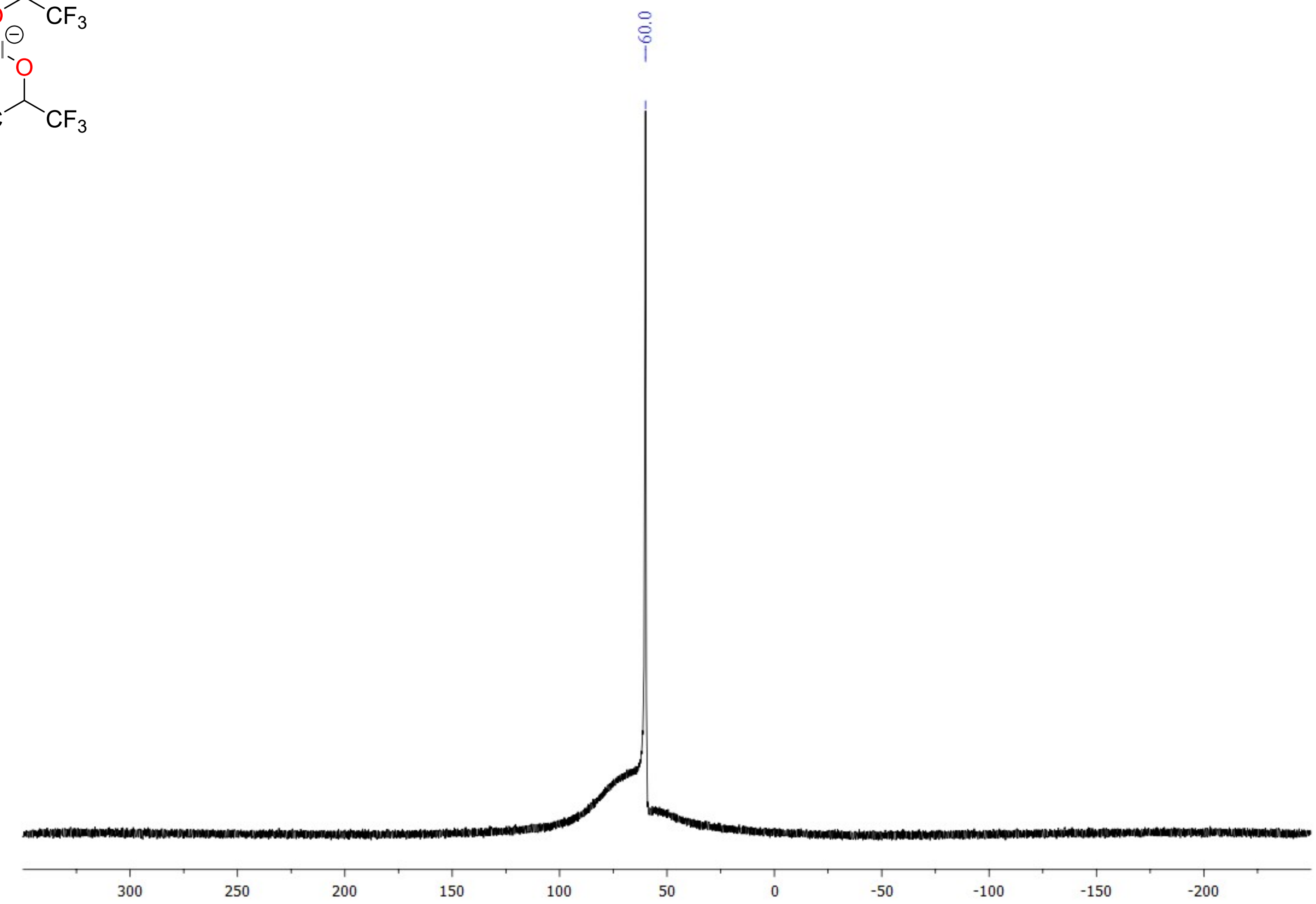

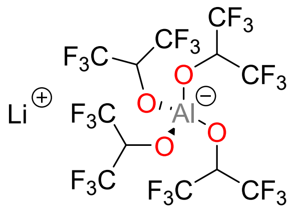

Figure S8.1.14 ^{19}F NMR (376 MHz, CD_3CN, 295 K) spectrum of lithium tetrakis(hexafluoroisopropoxy)aluminate, Li[Al(hfip)$_4$]·DME (**1b**).

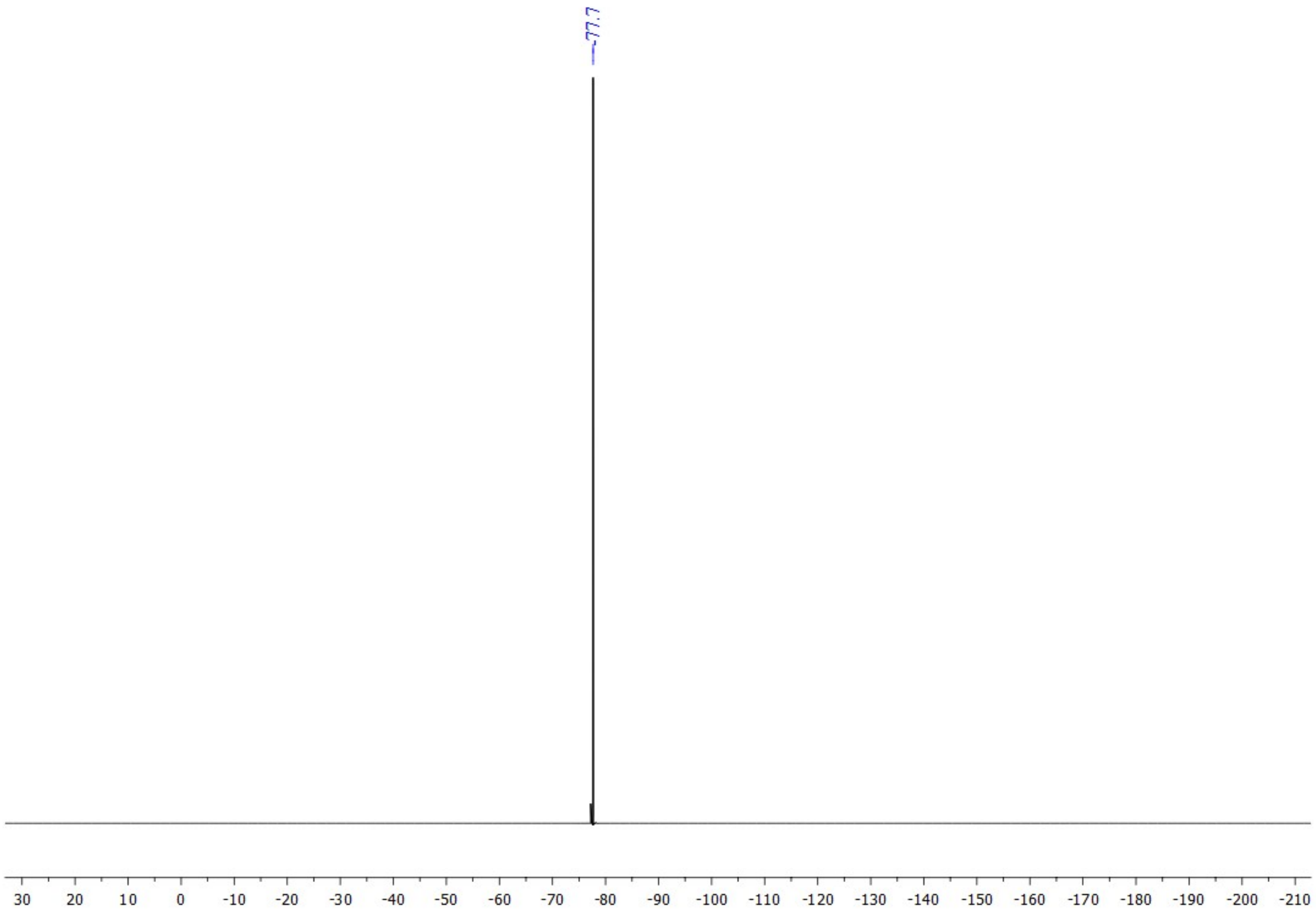

Figure S8.1.15 ^{7}Li NMR (155 MHz, CD_3CN, 295 K) spectrum of lithium tetrakis(hexafluoroisopropoxy)aluminate, Li[Al(hfip)$_4$] (**1b**).

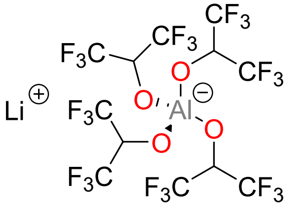

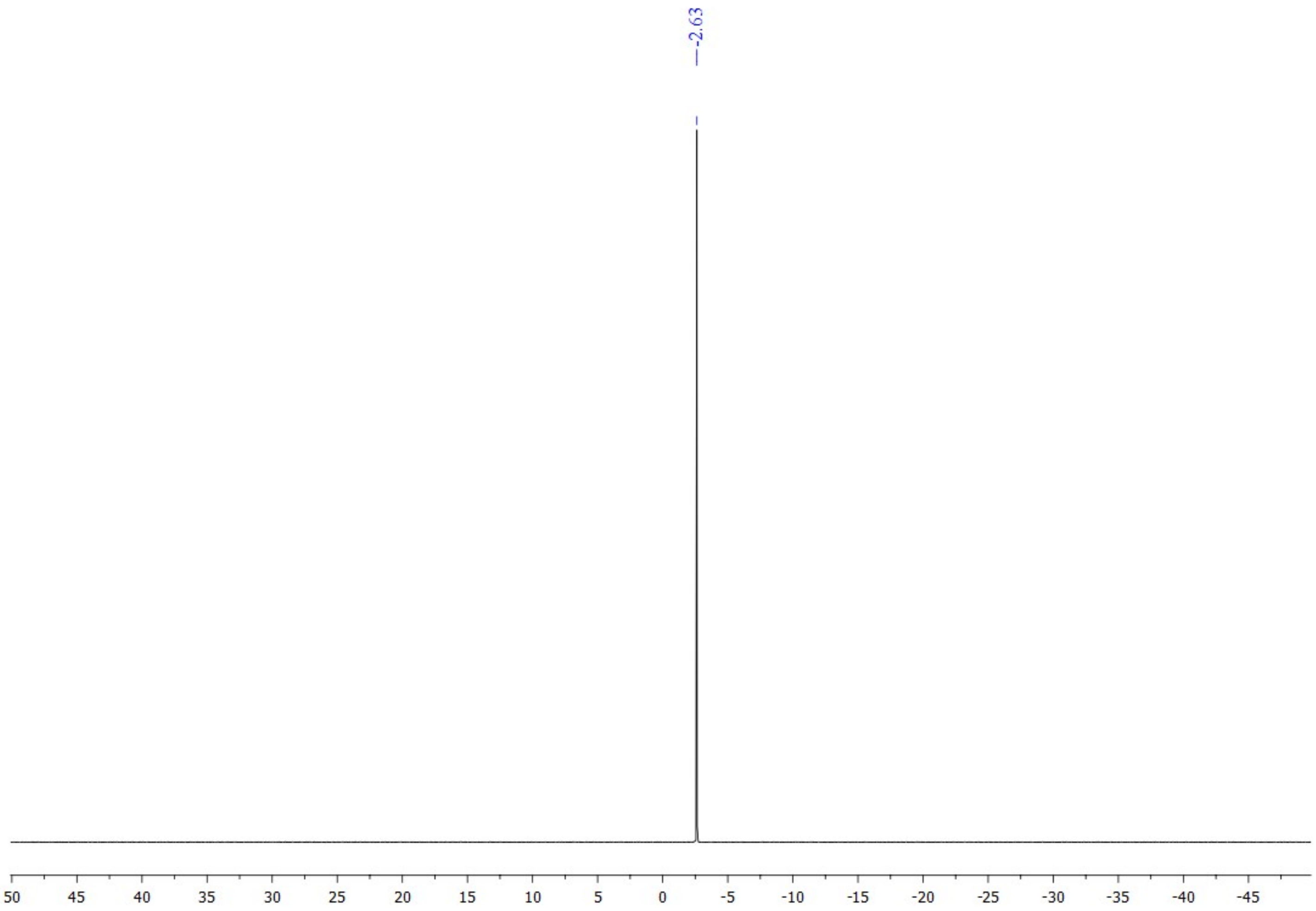

-2.63
50
45
40
35
30
25
20
15
10
5
0
-5
-10
-15
-20
-25
-30
-35
-40
-45

S8.2 NMR spectra of air exposed lithium borate and aluminate complexes

Figure S8.2.1 ^1H NMR (400 MHz, $(CD_3)_2SO$, 295 K) spectrum of lithium tetrakis(hexafluoroisopropoxy)borate, Li[B(hfip)$_4$]·2DME (**1a**·2DME) after 24 hour air exposure.

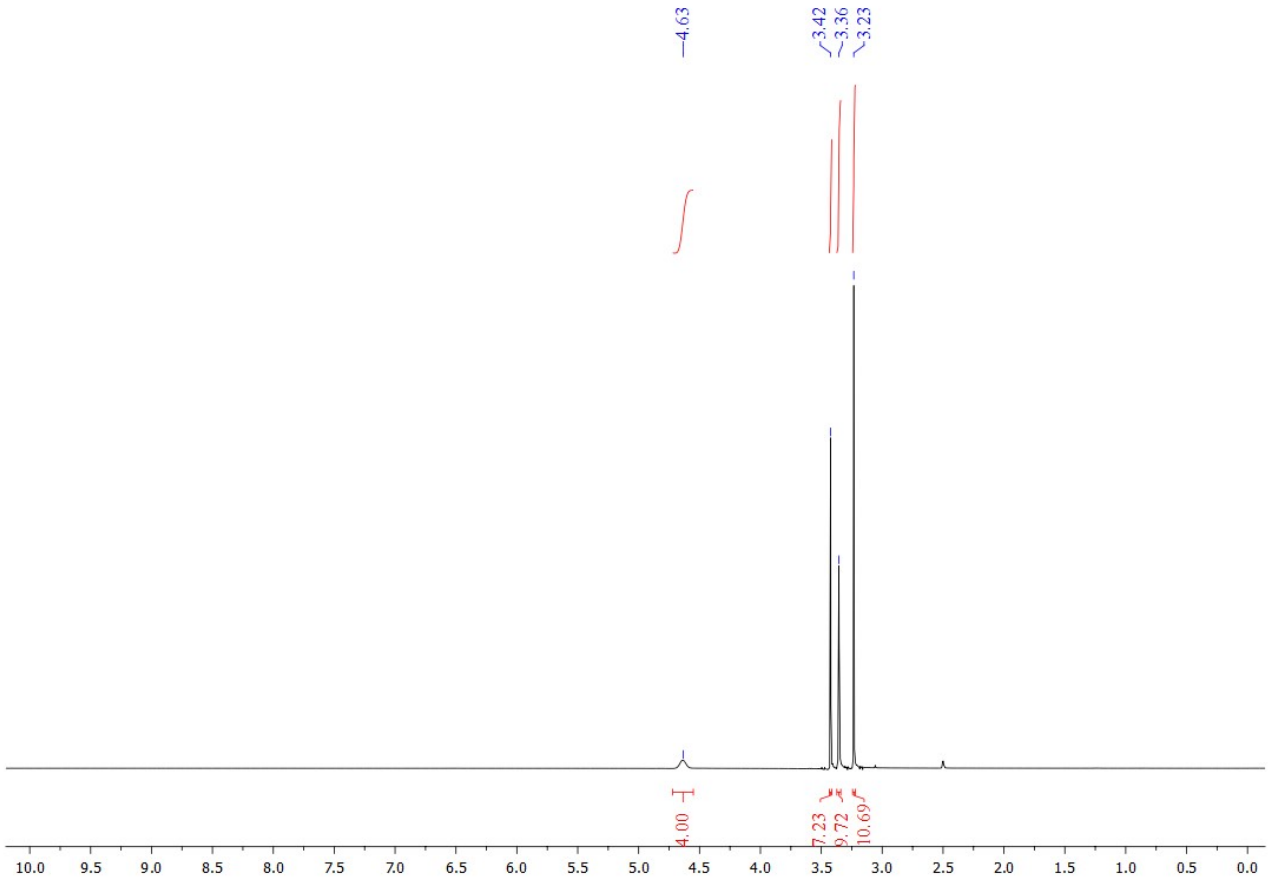

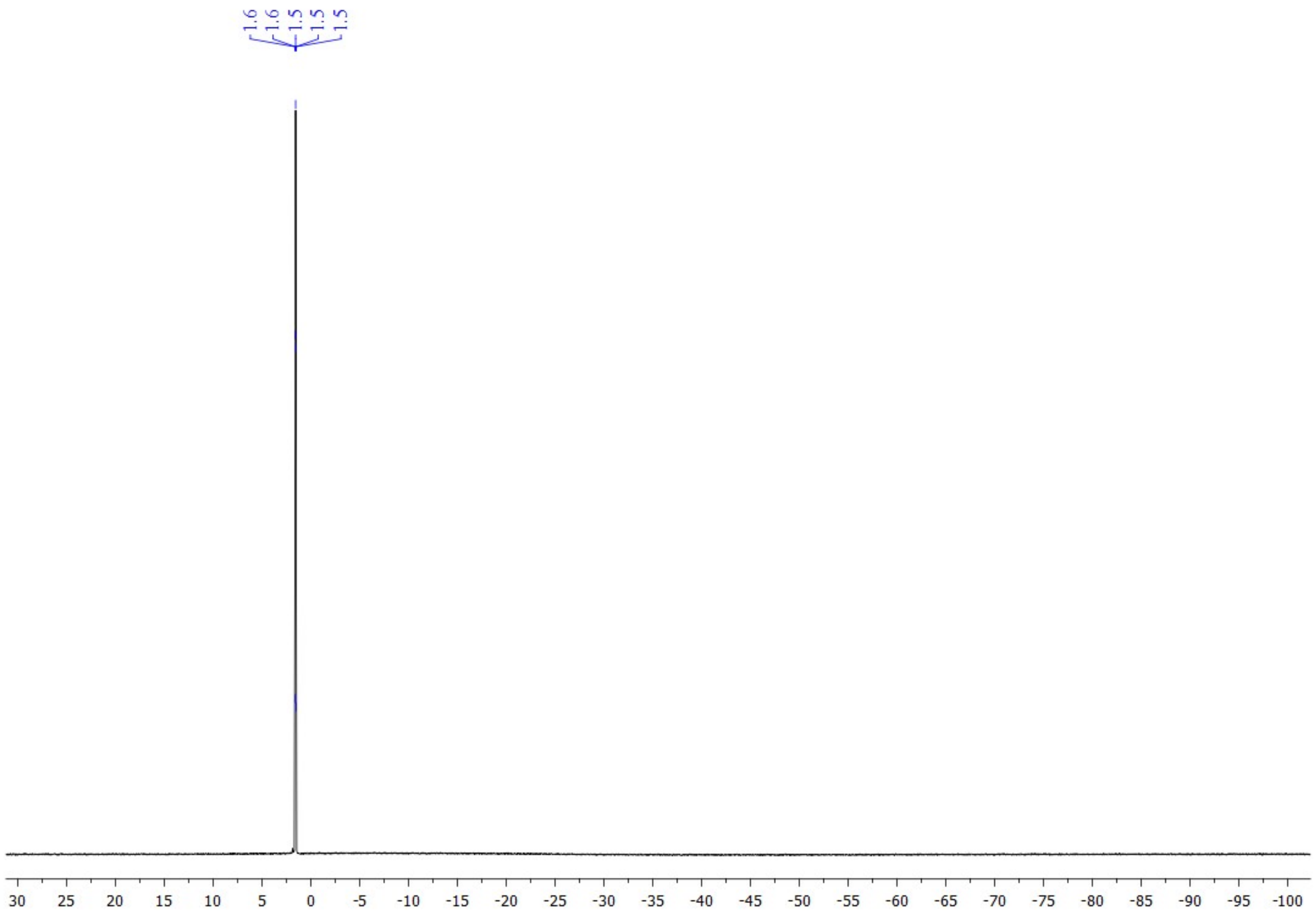

Figure S8.2.2 ^{11}B NMR (128 MHz, $(CD_3)_2SO$, 295 K) spectrum of lithium tetrakis(hexafluoroisopropoxy)borate, Li[B(hfip)$_4$]·2DME (**1a**·2DME) after 24 hour air exposure.

Figure S8.2.3 ^{19}F NMR (376 MHz, $(CD_3)_2SO$, 295 K) spectrum of lithium tetrakis(hexafluoroisopropoxy)borate, Li[B(hfip)$_4$]·2DME (**1a**·2DME) after 24 hour air exposure.

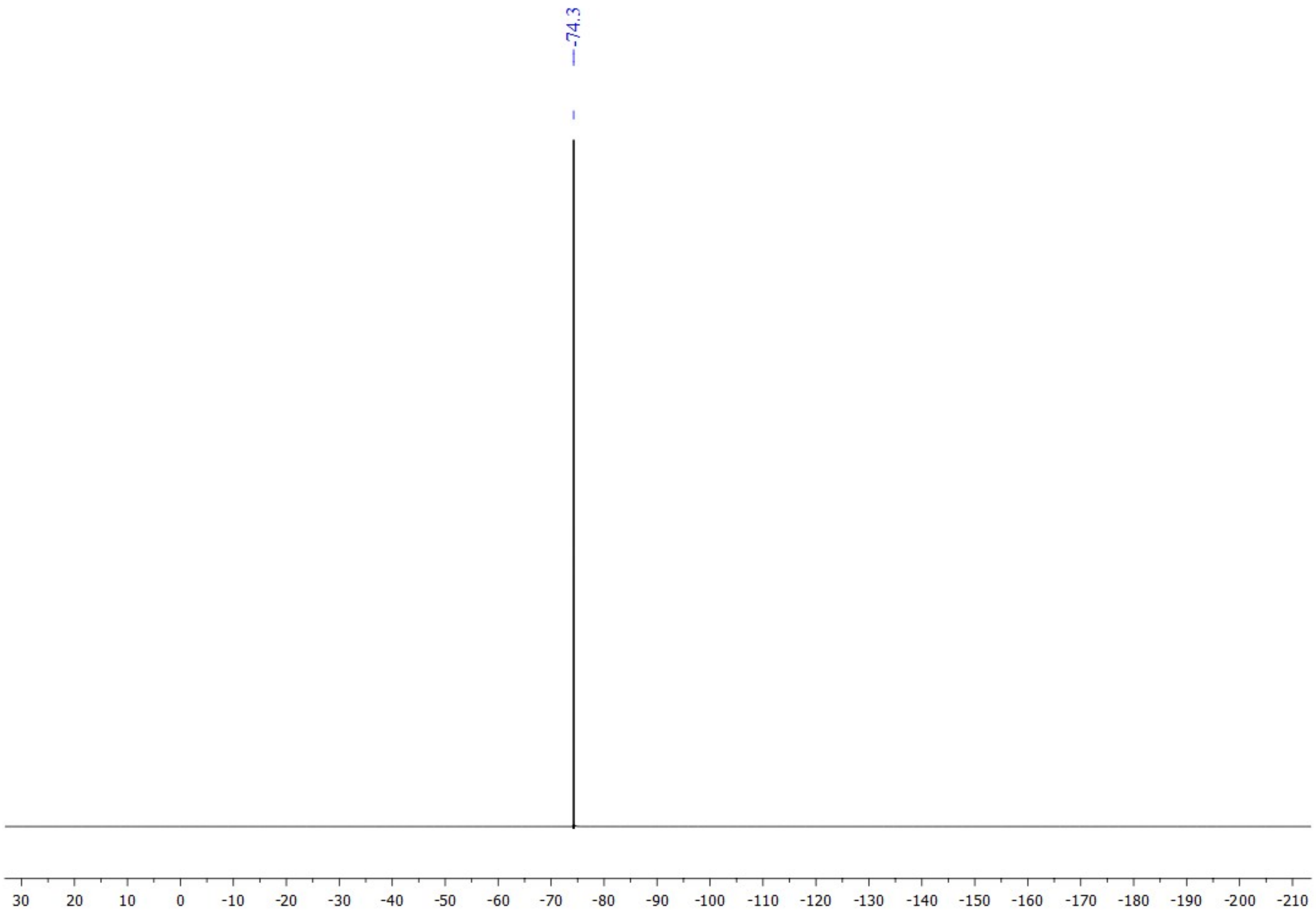

Figure S8.2.4 ^{7}Li NMR (155 MHz, $(CD_3)_2SO$, 295 K) spectrum of lithium tetrakis(hexafluoroisopropoxy)borate, Li[B(hfip)$_4$]·2DME (**1a**·2DME) after 24 hour air exposure.

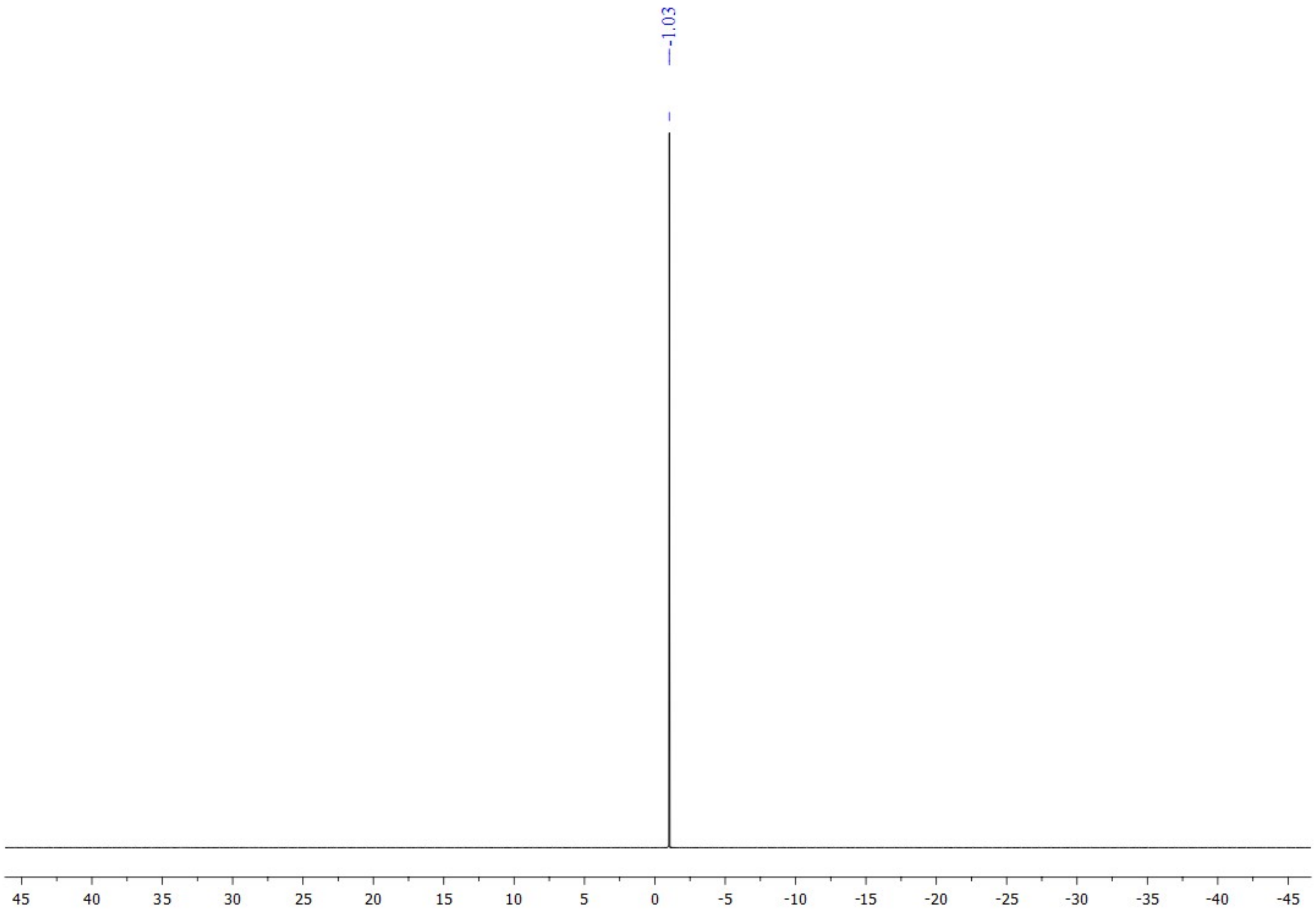

Figure S8.2.5 Top: ^{1}H NMR (400 MHz, $(CD_3)_2SO$, 295 K) spectrum of Li[B(hfip)$_4$]·2DME (**1a**·2DME) after 24 hour air exposure. Bottom: ^{1}H NMR (500 MHz, $(CD_3)_2SO$, 295 K) spectrum of pristine Li[B(hfip)$_4$]·2DME (**1a**·2DME). Signal at 3.36 ppm in the air exposed sample is assigned to water.

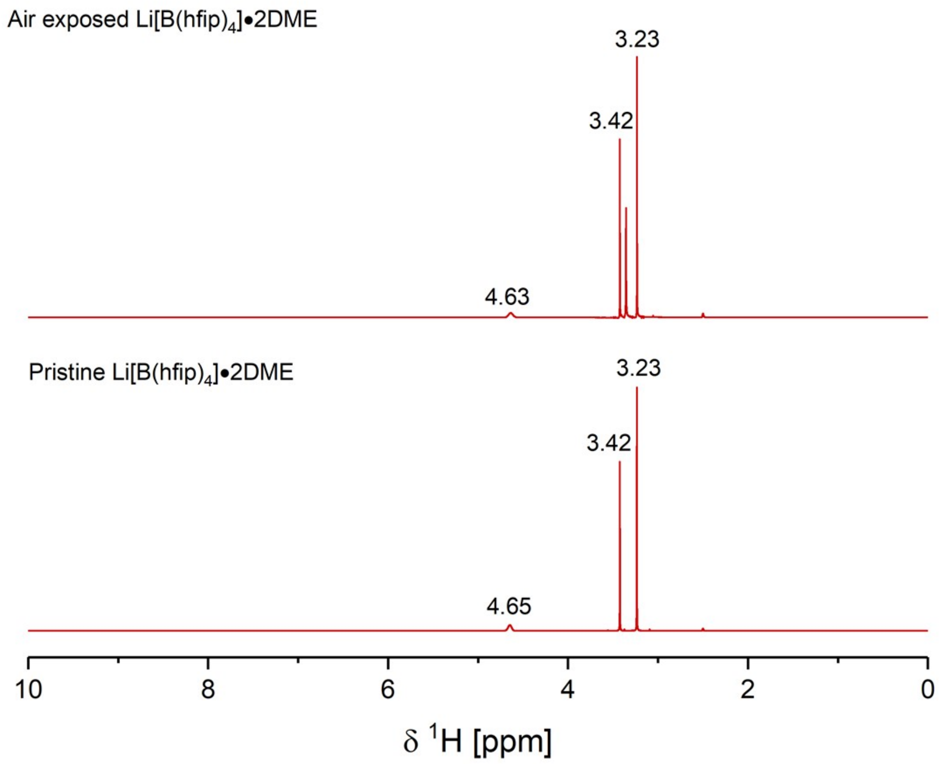

Figure S8.2.6 Top: ^{11}B NMR (128 MHz, $(CD_3)_2SO$, 295 K) spectrum of Li[B(hfip)$_4$]·2DME (**1a**·2DME) after 24 hour air exposure. Bottom: ^{11}B NMR (128 MHz, $(CD_3)_2SO$, 295 K) spectrum of pristine Li[B(hfip)$_4$]·2DME (**1a**·2DME).

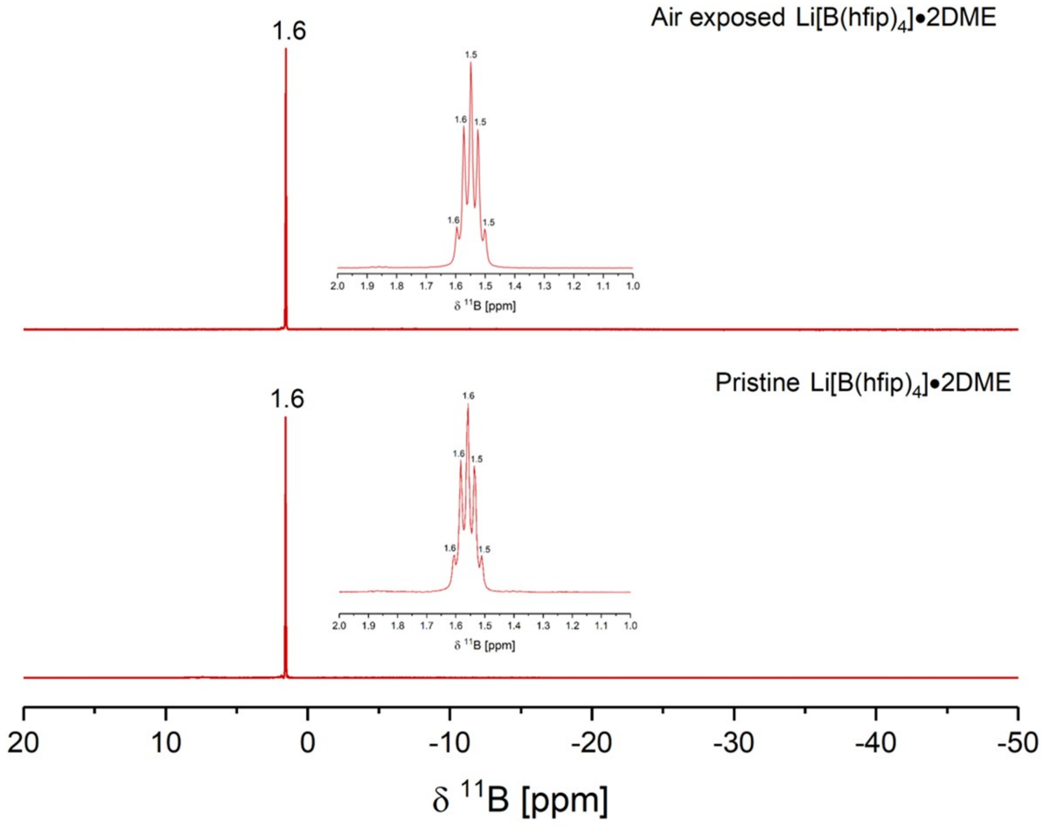

Figure S8.2.7 Top: ^{19}F NMR (376 MHz, $(CD_3)_2SO$, 295 K) spectrum of Li[B(hfip)$_4$]·2DME (**1a**·2DME) after 24 hour air exposure. Bottom: ^{19}F NMR (471 MHz, $(CD_3)_2SO$, 295 K) spectrum of pristine Li[B(hfip)$_4$]·2DME (**1a**·2DME).

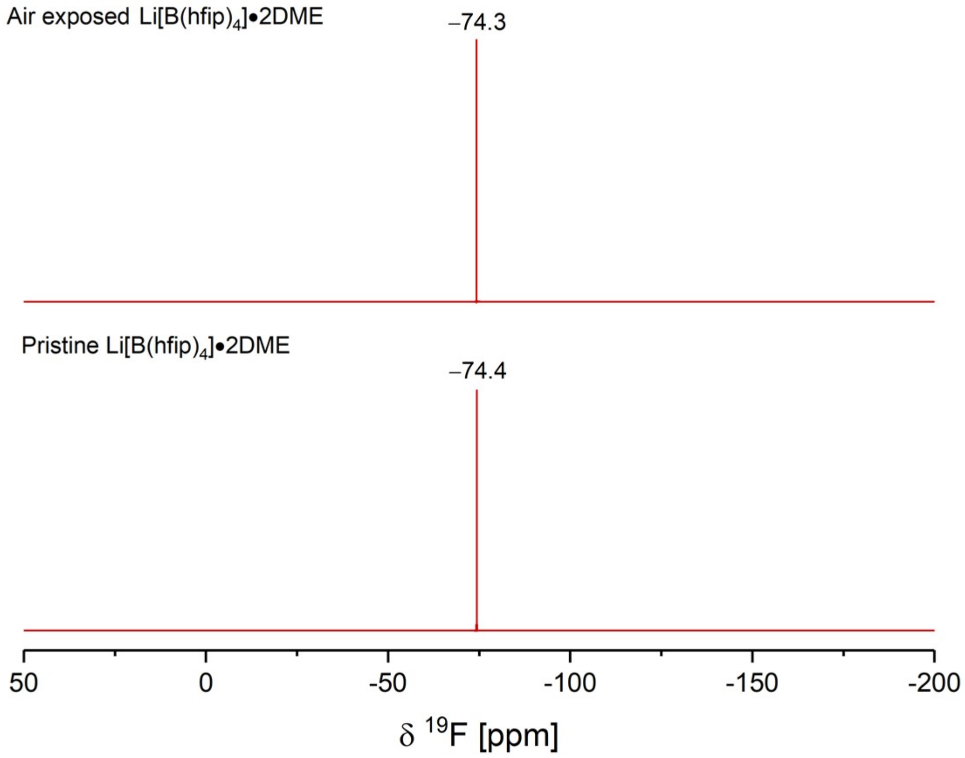

Figure S8.2.8 Top: ^{7}Li NMR (155 MHz, $(CD_3)_2SO$, 295 K) spectrum of Li[B(hfip)$_4$]·2DME (**1a**·2DME) after 24 hour air exposure. Bottom: ^{7}Li NMR (155 MHz, $(CD_3)_2SO$, 295 K) spectrum of pristine Li[B(hfip)$_4$]·2DME (**1a**·2DME).

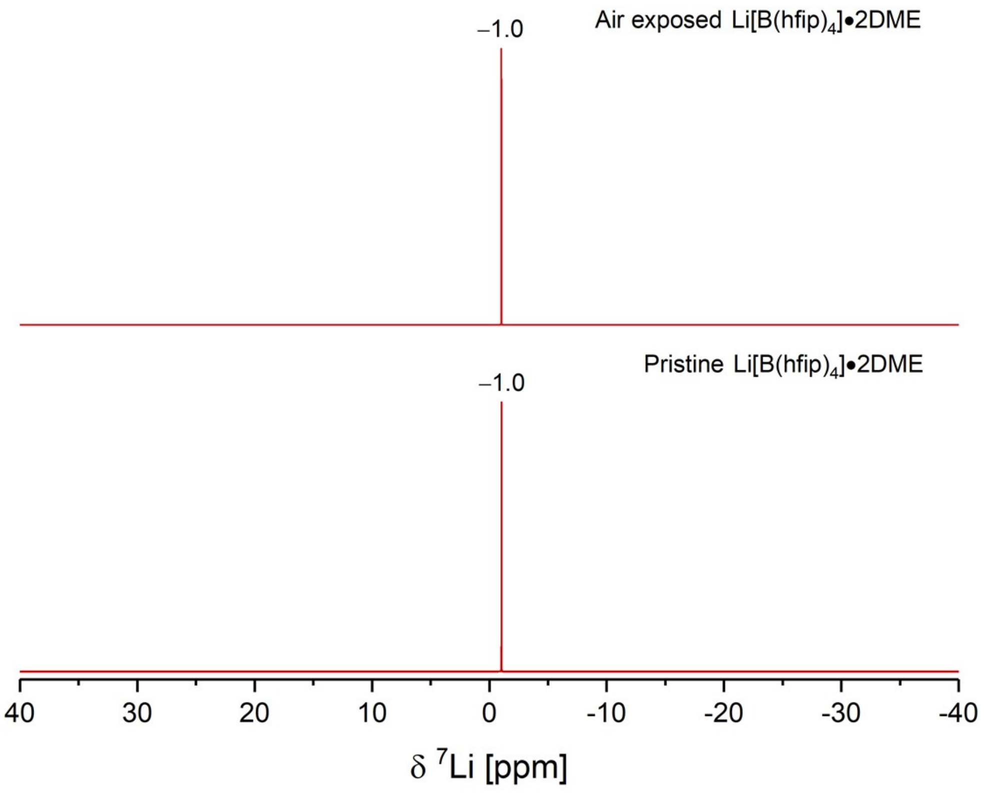

Figure S8.2.9 ^{1}H ssNMR (16.4 T, 40 kHz) spectrum of lithium tetrakis(hexafluoroisopropoxy)aluminate, Li[Al(hfip)$_4$]·DME (**1b**·DME) after 24 hour air exposure.

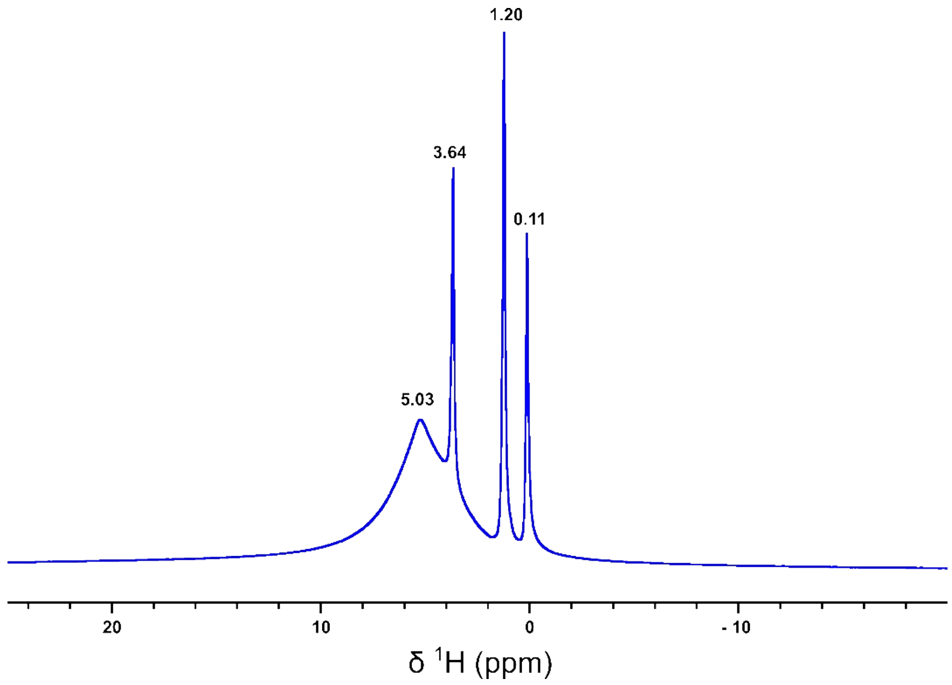

Figure S8.2.10 ^{19}F ssNMR (16.4 T, 40 kHz) spectrum of lithium tetrakis(hexafluoroisopropoxy)aluminate, Li[Al(hfip)$_4$]·DME (**1b**·DME) after 24 hour air exposure.

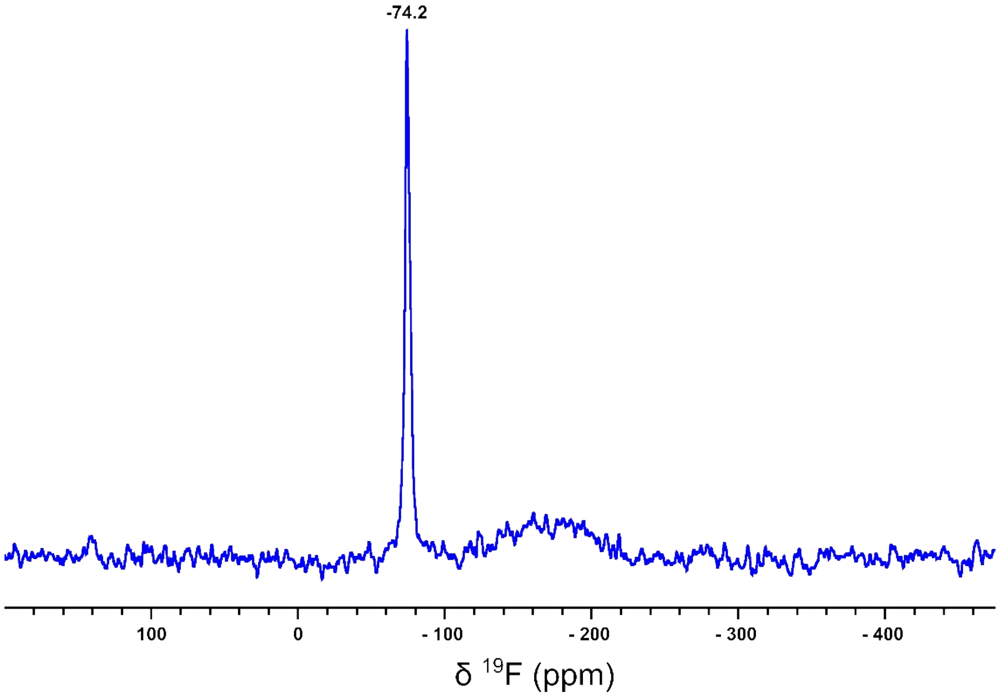

Figure S8.2.11 ^{27}Al ssNMR (16.4 T, 40 KHz) spectrum of lithium tetrakis(hexafluoroisopropoxy)aluminate, Li[Al(hfip)$_4$]·DME (**1b**·DME) after 24 hour air exposure.

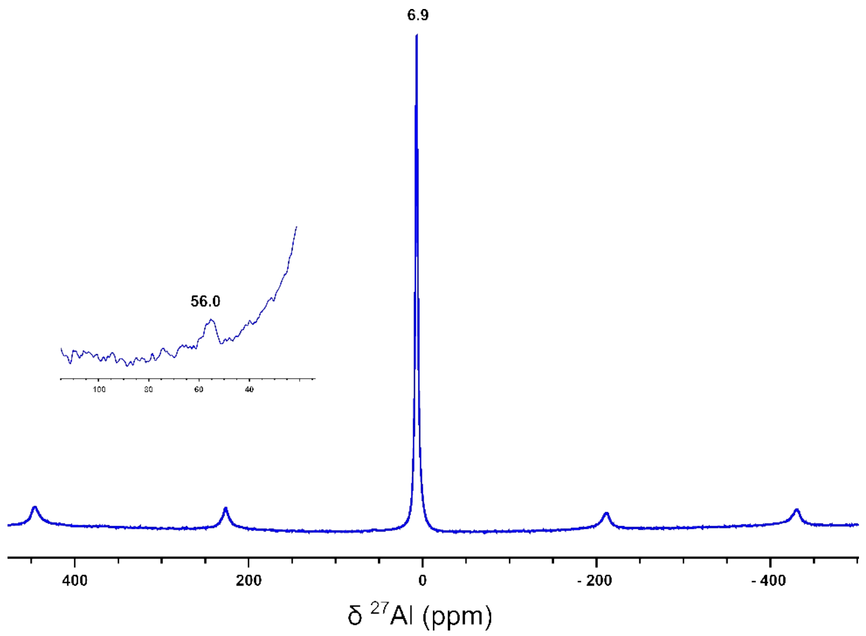

Supplement: CC-061-D4CC04812A-s001 [file CC-061-D4CC04812A-s001.pdf]
